# Impact of various high fat diets on gene expression and the microbiome across the mouse intestines

**DOI:** 10.1038/s41598-023-49555-7

**Published:** 2023-12-27

**Authors:** Jose Martinez-Lomeli, Poonamjot Deol, Jonathan R. Deans, Tao Jiang, Paul Ruegger, James Borneman, Frances M. Sladek

**Affiliations:** 1 Genetics, Genomics and Bioinformatics Graduate Program, University of California, Riverside, CA 92521 USA; 2 Department of Molecular, Cell and Systems Biology, University of California, Riverside, CA 92521 USA; 3 Department of Computer Science and Engineering, University of California, Riverside, CA 92521 USA; 4 Institute of Integrated Genome Biology, University of California, Riverside, CA 92521 USA; 5 Department of Microbiology and Plant Pathology, University of California, Riverside, CA 92521 USA

**Keywords:** Gastrointestinal system, Transcriptomics

## Abstract

High fat diets (HFDs) have been linked to several diseases including obesity, diabetes, fatty liver, inflammatory bowel disease (IBD) and colon cancer. In this study, we examined the impact on intestinal gene expression of three isocaloric HFDs that differed only in their fatty acid composition—coconut oil (saturated fats), conventional soybean oil (polyunsaturated fats) and a genetically modified soybean oil (monounsaturated fats). Four functionally distinct segments of the mouse intestinal tract were analyzed using RNA-seq—duodenum, jejunum, terminal ileum and proximal colon. We found considerable dysregulation of genes in multiple tissues with the different diets, including those encoding nuclear receptors and genes involved in xenobiotic and drug metabolism, epithelial barrier function, IBD and colon cancer as well as genes associated with the microbiome and COVID-19. Network analysis shows that genes involved in metabolism tend to be upregulated by the HFDs while genes related to the immune system are downregulated; neurotransmitter signaling was also dysregulated by the HFDs. Genomic sequencing also revealed a microbiome altered by the HFDs. This study highlights the potential impact of different HFDs on gut health with implications for the organism as a whole and will serve as a reference for gene expression along the length of the intestines.

## Introduction

Over the last several decades the average diet in the U.S. has become increasingly high in fat and low in fiber. There has also been a change in the type of fat consumed by Americans such that soybean oil, high in polyunsaturated fat (PUFA), is currently the predominant source of dietary fat^1^. High-fat diets (HFDs) have been linked to several diseases, including obesity, diabetes, insulin resistance, fatty liver and susceptibility to inflammatory bowel disease (IBD) in both mice and humans^2–5^. They also impact the gut microbiota^6,7^, physiological changes in the small intestine^8^, intestinal permeability and gastrointestinal diseases^9^. However, most gene expression studies analyze only one portion of the intestines or one type of HFD at a time^10–12^ and they typically use diets made with saturated animal fat, not plant-based unsaturated oils.

Here, we used RNA-seq to examine the impact of three HFDs on gene expression in four functionally distinct segments of the mouse intestinal tract: the duodenum, jejunum, terminal ileum, and proximal colon. The duodenum is responsible for breaking down the stomach acid and food mixture, while the jejunum absorbs sugars, amino acids, and fatty acids. The terminal ileum absorbs remaining nutrients, such as vitamin B12 and bile acids, and the proximal colon is the primary site for absorption of water and salts and microbial production of short chain fatty acids (SCFAs). All four parts of the intestine are also involved in xenobiotic and drug metabolism^13^.

The HFDs used in this study are comparable to the current American diet in that they consist of 40% of calories from fat and are low in fiber while most experimental HFDs use 50–60% kcal fat^14,15^. The first diet was formulated with coconut oil (saturated fat), the second with soybean oil (53% linoleic acid, LA, C18:2 omega-6) and the third with a genetically modified soybean oil with a fatty acid composition similar to olive oil (74% oleic acid C18:1, a monounsaturated fat, MUFA). Each diet was compared to a low fat (13% kcal fat), high-fiber vivarium chow as well as to each other. RNA-seq analysis revealed dysregulation of several nuclear receptor genes and other transcriptional regulators as well as xenobiotic/drug metabolism genes throughout the small and large intestines. There was significant dysregulation of genes involved in epithelial barrier function, IBD and colon cancer. Network analysis showed an upregulation in metabolism genes and, interestingly, a downregulation in numerous genes involved in the immune system, particularly those related to bacterial and viral infections, including SARS-CoV-2, the pathogen responsible for the global COVID-19 pandemic. Finally, the expression of several genes related to signaling by neurotransmitters and the microbiome was dysregulated while genome sequencing revealed alterations in the gut bacteria by the HFDs.

## Materials and methods

### Animals

Care and treatment of animals was in accordance with guidelines from and approved by the University of California, Riverside Institutional Animal Care and Use Committee (AUP #20140014). All animals were treated as previously described^3^ and in accordance with ARRIVE guidelines. Briefly, male C57BL/6N mice weaned at three weeks of age were assigned randomly to one of four diets for 24 weeks—low fat (13% kcal) Vivarium (VIV) chow; coconut oil (CO, 36% kcal from coconut oil and 4% kcal from soybean oil to provide the essential fatty acids LA and alpha-linolenic acid, ALA); CO plus soybean oil (SO + CO, 21% kcal from coconut oil and 19% kcal from soybean oil, resulting in 10% kcal from LA, comparable to the amount in the current American diet^16^); CO plus Plenish soybean oil (PL + CO, as SO + CO but with conventional soybean oil replaced on a per gram basis with the genetically modified High Oleic Soybean Oil Plenish [DuPont Pioneer, Johnston, IA] resulting in 1.4% kcal LA and 14% kcal oleic acid)^17^ (see Supplementary Table S1 for a comparison of the diets and Deol et al.^3^ for the complete composition of the diets). Metabolic parameters of the mice, including body weight, glucose tolerance, insulin resistance and fatty liver were reported previously^3^. At the end of the study, animals were euthanized by CO_2_ inhalation followed by cervical dislocation. Intestinal tissue was excised immediately and put in RNALater for 24 h at room temperature and then stored at − 80 °C.

### RNA-seq

The tissues for RNA sequencing (RNA-seq) were duodenum (DUO, 1 cm immediately downstream of the gastroduodenal junction), jejunum (JEJ, 1 cm at the approximate middle of the remainder of the small intestine), terminal ileum (TI, 1 cm immediately upstream of the ileo-cecal junction), and proximal colon (PC, 1 cm immediately downstream of the ileo-cecal junction). Total RNA was isolated from each tissue (DUO, JEJ, TI and PC) using a miRNeasy kit (Qiagen, Inc., Valencia, CA) and evaluated by NanoDrop (Wilmington, DE) and Agilent 2100 Bioanalyzer (Santa Clara, CA) as previously described^2^. Poly(A) + RNA (4 μg) with an RNA Integrity Number (RIN) of 7.8 or higher was used to construct sequencing libraries with the TruSeq Long RNA Sample Prep Kit (Illumina, San Diego, CA). RNA libraries were validated for RNA integrity by Bioanalyzer, pooled in equimolar amounts, and sequenced on an Illumina HiSeq 2000 at the UCR Genomics Core to generate 50 bp paired-end reads. Three biological replicates were sequenced for the Vivarium Chow diet (VIV) and four for each of the three HFDs (CO, SO + CO, and PL + CO). On average ~ 16 million reads were acquired for each biological replicate. The raw data are publicly available in Gene Expression Omnibus (GEO), accession number GSE220302.

### Differential gene expression analysis of RNA-seq data

Reads were aligned to the mouse reference genome (mm10) with STAR v2.5.0a using default parameters^18^. Raw read counts were calculated with STAR using the GeneCounts option of the quantMode parameter since the libraries were unstranded. Library normalization was performed with EDASeq^19^; within-lane normalization on GC content was performed with the LOESS method and between-lane normalization was performed with the non-linear full quantile method. Normalization factors from EDASeq were used for differential expression analysis with DESeq2^20^. Normalized read counts, FPKM (fragments per kilobase per million), and r-log (regularized log transformation) results were generated for downstream analysis.

The list of genes used in the heatmaps for nuclear receptors, epithelial barrier, IBD, colon cancer, microbiome and COVID-19 were obtained from the NCBI website (Supplementary Table S6). Differentially expressed genes (DEGs) between any two diets (p-adj ≤ 0.05) were identified in the RNA-seq data and displayed in the respective heatmaps, generated using the Pheatmap package in R^21^ and row-normalized before plotting, unless noted otherwise. Python library “Plotly” was used to generate scatter plots for individual genes^22^. PCA analysis, bar plots and Venn diagrams were created using the Python library ‘matplotlib’. Volcano plots were generated using the ggplot2 package from R^23^. Colored spots are DEGs with (p-adj ≤ 0.05 and abs(Log2FC) ≥ 0.05); genes in the top 95% of − Log10(p-adj) and abs(Log2FC) ≥ 1.5 are indicated. StringApp^24^ from Cytoscape (Version 3.8.2)^25^ was used to analyze and visualize potential interactions between DEGs among the different diets and tissues in the KEGG^26^ and Reactome pathways^27^ (FDR ≤ 0.05); a medium interaction score of 0.4 (out of 0 to 1) in the StringApp was required. Mouse Genome Informatics (MGI) and GeneCards: The Human Gene Database were used to identify the full name of a gene, as well as function and associated diseases^28,29^.

### Microbiome analysis

The bacterial collection protocol, DNA extraction and bacterial rRNA internal transcribed spacer (ITS) analysis was performed as previously described^30^ except that bacteria were collected from the small intestine or colon of male mice fed the different diets (VIV, SO, SO + CO, PL, PL + CO) for 24 weeks—the same ones used for the RNA-seq. Only the top 12 genus-level of operational taxonomic units (OTU) were plotted as mean percentage compositions for each treatment group; the remaining OTUs were combined under "Other". DNA sequencing data of the microbiome is publicly available at SRA BioProject, Accession #PRJNA615924.

## Results

**Figure 1:**
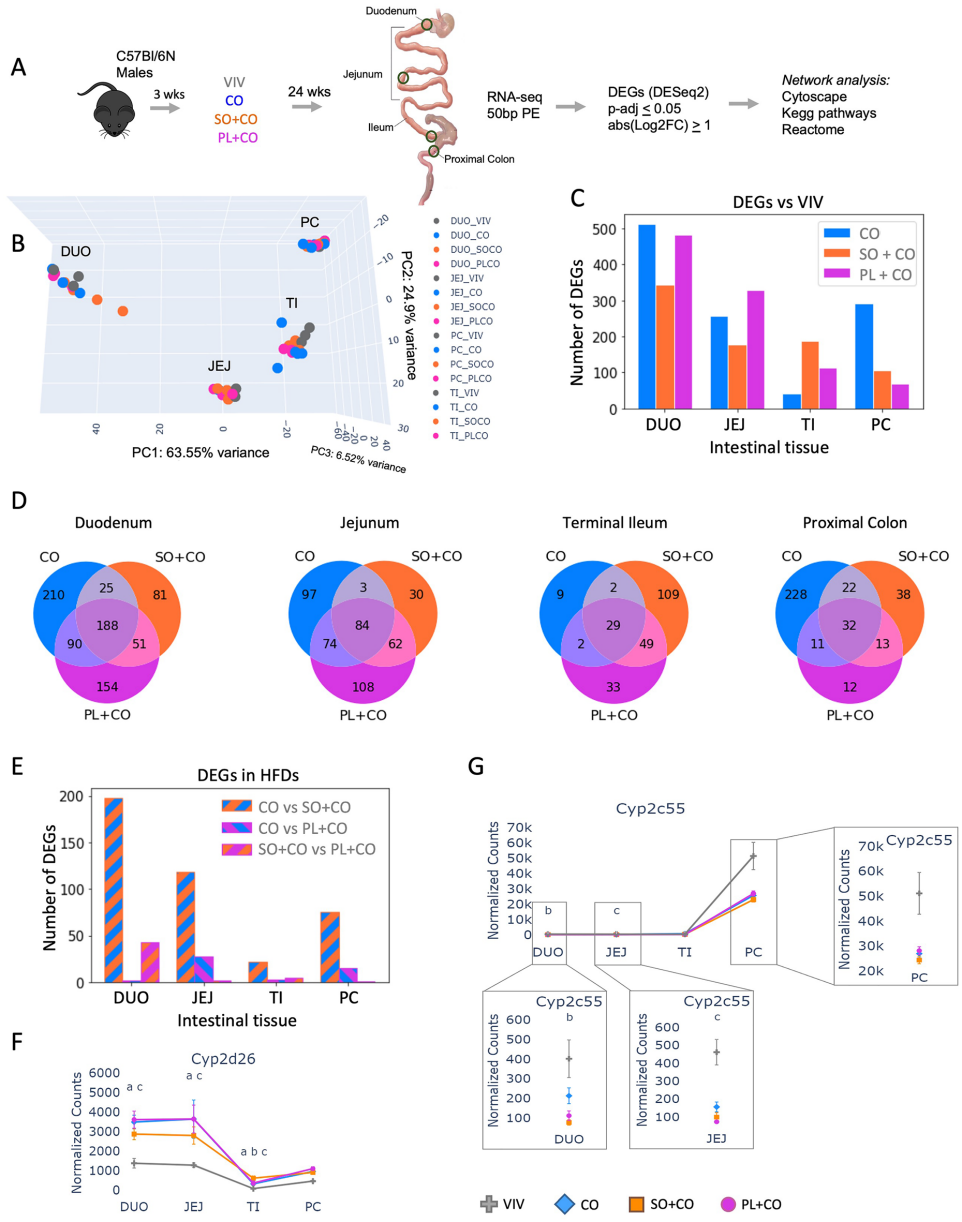
Differential impact of HFDs on gene expression across different parts of the intestines. (**A**) Work-flow: male C57Bl/6N male mice were weaned at 3 weeks of age to either a regular chow diet (VIV) or one of the three high fat diets—*CO* coconut oil, *SO* + *CO* soybean oil enriched, *PL* + *CO* low-LA soybean oil (Plenish) enriched. One centimeter of each tissue was used to perform RNA-seq (regions indicated with a circle). Post sequencing analysis was done as indicated. N = 3 per tissue for VIV and 4 per tissue for the HFDs. See Supplementary Table S1 for diet composition. (**B**) 3D principal component analysis (PCA) showing differential effects of the diets on different parts of the intestines. (**C**) Bar plot showing the number of differentially expressed genes (DEGs, up and down regulated) (p-adj ≤ 0.05 and absolute fold change ≥ 2 (abs(Log2FC) ≥ 1)) in three HFDs vs VIV chow in different parts of the intestines. See Supplementary Tables S2–S5 for complete comparison of genes between diets and Supplementary Fig. S2 for volcano plots of the most dysregulated genes. (**D**) Venn diagrams showing the overlap of the DEGs (p-adj < 0.05, Log2FC ≥ 1) in the indicated diet comparisons across the tissues. See Supplementary Fig. S1 for Venn analysis between HFDs. (**E**) Bar plot showing the number of differentially expressed genes (DEGs, up and down regulated) (p-adj ≤ 0.05 and absolute fold change ≥ 2 (abs(Log2FC) ≥ 1) between the three HFD comparisons in different parts of the intestines. (**F**,**G**) Line graph of the average normalized read counts with standard deviation (SD) of *Cyp2d26* (**F**) and *Cyp2c55* (**G**) in various parts of the intestines on the indicated diets (VIV, CO, SO + CO, PL + CO). Significantly different levels of expression between the diets within a given tissue denoted by p-adj ≤ 0.05 and are indicated as follows: a (VIV vs CO); b (VIV vs SO + CO); c (VIV vs PL + CO); d (CO vs SO + CO); e (CO vs PL + CO); f (SO + CO vs PL + CO). See Supplementary Figs. S3 and S4 for heatmaps and line graphs of additional *Cyp*, *Gst* and *UGT* genes.

Male C57BL/6N mice were fed one of four diets for a period of 24 weeks and gene expression was examined in different portions of the intestines (Fig. 1A). The diets included a low-fat Vivarium chow (VIV) and three high-fat diets (HFDs) with 40% of calories derived from different plant oils: coconut oil (CO), conventional soybean oil (SO + CO) and genetically modified soybean oil low in LA and high in oleic acid (PL + CO) (Supplementary Table S1). Previous analysis of these mice revealed that the soybean oil diet (SO + CO), and to a lesser extent the Plenish diet (PL + CO), induced obesity, diabetes, insulin resistance, and fatty liver, while the isocaloric CO diet had minimal adverse metabolic effects despite similar caloric intake as the other HFDs^3^. RNA-seq was performed on a segment of each of the four tissues: duodenum (DUO), jejunum (JEJ), terminal ileum (TI), and proximal colon (PC). Differentially expressed genes (DEGs) were defined as having a p-adjusted value of less than 0.05 and an absolute fold change greater than 2 (p-adj < 0.05 and Log2FC > 1.0). The DEGs (p-adj < 0.05) were further analyzed using network analysis in Cytoscape and the KEGG and Reactome databases (Fig. 1A).

### HFDs alter gene expression in a differential fashion across the intestinal tract, including drug metabolism genes

Principal Components Analysis (PCA) of the 60 RNA-seq datasets revealed that the transcriptomes were primarily grouped based on tissue, with smaller variations observed between dietary groups (Fig. 1B). Nonetheless, a considerable number of DEGs were identified when any of the three HFDs were compared to the VIV chow within a specific tissue (Fig. 1C). The duodenum (DUO) exhibited the greatest number of DEGs in all three HFD vs. VIV chow comparisons (CO: 513; SO + CO: 345; PL + CO: 483). The jejunum (JEJ) also had a substantial number of DEGs, albeit fewer than the duodenum (CO: 258; SO + CO: 179; PL + CO: 328), while the terminal ileum (TI) had a lower number of DEGs, except for the SO + CO vs. VIV chow (CO: 42; SO + CO: 189; PL + CO: 113). In contrast, the proximal colon (PC) displayed the largest number of DEGs in the CO vs. VIV comparison (CO: 293; SO + CO: 105; PL + CO: 68) (Fig. 1C). A Venn analysis revealed a moderate to minimal overlap in DEGs between the different HFDs and the VIV chow, ranging from 188 genes in the duodenum to 29 genes in the terminal ileum (Fig. 1D). These findings indicate that diets composed of different fats have distinct impacts on specific segments of the intestines.

Comparison between each of the three HFDs showed that CO vs. SO + CO consistently yielded the greatest number of DEGs (DUO: 198; JEJ: 118; TI: 22; PC: 75) (Fig. 1E). In contrast, CO vs. PL + CO exhibited a surprisingly low number of DEGs (ranging from 2 to 28) in all four tissues, except for the duodenum, which had 43 DEGs. Venn analysis of the pairwise comparisons between the HFDs revealed no overlap in DEGs among all three comparisons and relatively limited overlap between any two comparisons (Supplementary Fig. S1).

Volcano plot analysis identified individual genes with significant fold change in various HFD vs. VIV chow comparisons, including several cytochrome P450 (*Cyp*) genes (Supplementary Fig. S2). For example, *Cyp2d26* was expressed at higher levels in the small intestines than the proximal colon and significantly upregulated by all three HFDs (Fig. 1F). In contrast, *Cyp2c55* was expressed at much higher levels in the proximal colon than the small intestines and the HFDs tended to decrease expression, although it did not reach significance (Fig. 1G). Several other *Cyp* genes (*Cyp4a10*, *Cyp4a31*, *Cyp4a32, Cyp4f15, Cyp2j6, Cyp2j9*) were upregulated primarily in the duodenum by all three HFDs while a few genes were dysregulated in the jejunum by one or more HFDs (*Cyp2u1*, *Cyp2c29*, *Cyp4f16*) (Supplementary Fig. S3). Expression of other *Cyp* genes as well as Phase 2 *Ugt* and *Gst* genes also varied across the intestines on the VIV chow and in response to the different HFDs, with a very modest impact on relatively few Phase 2 genes (e.g., *Gstm1*, *Gsta4*, *Ugt1a9*, *Ugt1a7*, *Ugt2b36*) and a greater impact on a number of *Cyp* genes (Supplementary Fig. S4).

### Differential expression of nuclear receptors across the intestinal tract and in response to HFD

**Figure 2:**
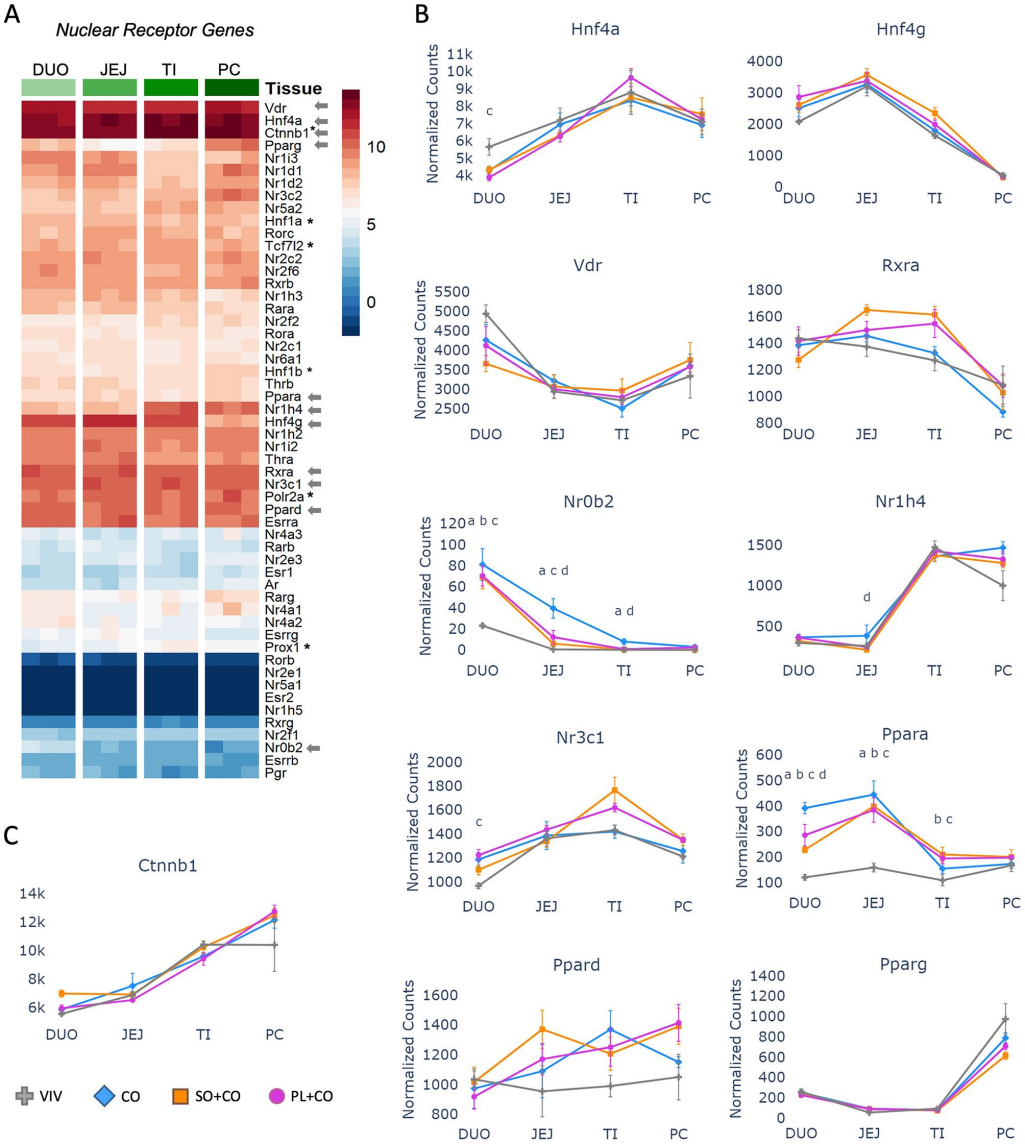
Differential expression of nuclear receptors across the intestinal tract and in different HFDs. (**A**) Non row-normalized heatmap showing levels of all 48 nuclear receptors (NR) across the tissues in mice fed VIV chow, sorted by levels in the duodenum (DUO) and compared to non NR transcription factors (*). Normalized read counts across three biological replicates are shown. *JEJ* Jejunum, *TI* Terminal Ileum, *PC* Proximal colon, *Arrows* genes plotted in figure. Arbitrary scale of relative expression is shown. See Supplementary Fig. S5 for additional heatmaps of nuclear receptors. (**B**) Line graphs showing normalized read counts with standard deviation (SD) of select NRs in various parts of the intestines on the indicated diets. Significantly different genes between diets within a given tissue (p-adj ≤ 0.05) are indicated as follows: a (VIV vs CO); b (VIV vs SO + CO); c (VIV vs PL + CO); d (CO vs SO + CO). (**C**) As in (**B**) but for beta-catenin (*Ctnnb1*).

Several members of the nuclear receptor (NRs) superfamily of ligand-dependent transcription factors are known to regulate CYP genes and play important roles in the development and function of the intestinal tract, as well as pathologies such as IBD and colon cancer^31,32^. To determine their relative expression in different parts of the intestines we compared all 48 NRs across the four intestinal tissues in the mice fed VIV chow in a non row-normalized heatmap and included several non-NR transcription factors (TFs) known to play a role in intestinal physiology (*Ctnnb1, Hnf1a, Hnf1b, Polr2a, Prox1, Tcf7l2*) as a point of reference. The most highly expressed NR gene throughout the intestines is hepatocyte nuclear receptor 4 alpha (*Hnf4a*)—its expression was greater than that of RNA polymerase 2 (*Polr2a*) and nearly as high as beta-catenin (*Ctnnb1*)—followed by the vitamin D3 receptor (*Vdr*), *Hnf4g*, and *Rxra* (Fig. 2A). This relative order was maintained across the three HFDs as well (Supplementary Fig. S5). Some NR genes (e.g., *Hnf4a*, *Nr1h4*, *Pparg*) are expressed at lower levels in duodenum or jejunum, and at higher levels further along the intestinal tract while others (e.g., *Hnf4g*, *Vdr, Nr0b2*, and *Ppara*) have a relatively high level of expression in the beginning of the intestines and then decrease in the latter portions (Fig. 2B,C). Others, such as *Rxra,* which is a heterodimeric partner for many other NRs, have a fairly consistent level of expression across the four tissues, decreasing only in the proximal colon (Fig. 2B).

Among the top four most highly expressed NRs, the only one that showed differential expression among the different diets was *Hnf4a*. Its expression in the duodenum was decreased in mice fed any of the three HFDs compared to VIV chow (Fig. 2B). *Nr0b2* (short heterodimeric partner, SHP) which acts as a transcriptional repressor, the bile acid receptor (FXR, *Nr1h4*) and the glucocorticoid receptor (GR, *Nr3c1*), which plays a critical role in the stress response, all showed a significant difference from VIV chow in one or more HFD in at least one portion of the intestines (Fig. 2B). In contrast, there was no significant difference in *Ctnnb1* expression among the various diets, which is noteworthy as both HFD and mutations in the Wnt-Beta-catenin pathway are risk factors for colon cancer in humans (Fig. 2C)^33^.

Finally, we examined the PPARs, which are known to play a role in the regulation of nutrient transport from the lumen into the body and have fatty acids as their ligands. While *Ppard* and *Pparg* did not show any significant difference in expression between diets within a given tissue, *Ppara* expression was significantly increased in the duodenum and jejunum in all three HFDs. It was also increased in CO vs PL + CO in the duodenum and in SO + CO or PL + CO vs VIV chow in the terminal ileum (Fig. 2B).

### HFD impacts the expression of intestinal epithelial barrier function genes

**Figure 3:**
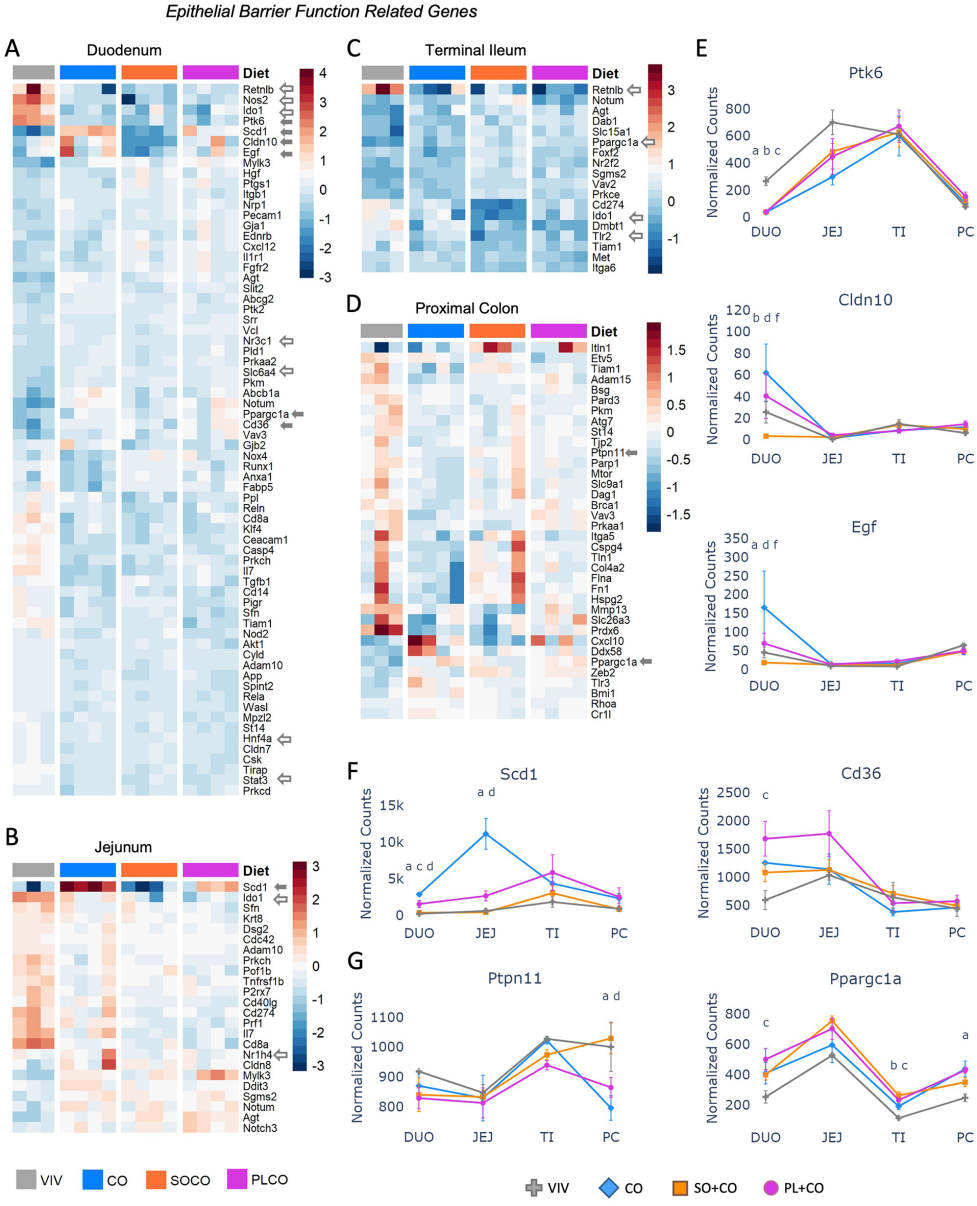
HFDs impact the expression of epithelial barrier function genes across the intestines. (**A**–**D**) Heatmaps of genes involved in epithelial barrier function in the indicated portions of the intestines of mice fed either low fat VIV chow or one of the three HFDs. Included are genes that are significantly different between any two diets (p-adj ≤ 0.05). Solid arrow, plotted in figure; open arrow, plotted in a subsequent figure. Arbitrary scale of relative expression is shown. See Supplementary Table S6 for a complete list of genes. (**E**–**G**) Line graphs showing normalized read counts with standard deviation (SD) of select genes on the indicated diets. Genes with significantly different levels of expression between the diets within a given tissue (p-adj ≤ 0.05) are indicated as follows: a (VIV vs CO); b (VIV vs SO + CO); c (VIV vs PL + CO); d (CO vs SO + CO); f (SO + CO vs PL + CO).

Formation and maintenance of a healthy epithelial barrier is an important physiological function of the intestines. To analyze the effect of diet on intestinal barrier function, we used a list of 444 genes from NCBI (Supplementary Table S6) and identified 123 genes that are significantly dysregulated (p-adj < 0.05) between any two dietary groups (Fig. 3A–D). The duodenum had the greatest number of dysregulated genes (mostly downregulated) across the different diets (68 genes). Several genes exhibited lower levels of expression in one or more HFDs compared to the VIV chow in the duodenum—e.g., *Ptk6* (Protein tyrosine kinase 6), *Cldn10* (Claudin 10), *Egf* (epidermal growth factor). In contrast, *Cd36* (cluster of differentiation 36) showed increased expression in PL + CO vs VIV chow in the duodenum while NR co-activator *Ppargc1a* (PPARG Coactivator 1 Alpha) showed elevated expression in one or more HFD in all parts of the intestines except the jejunum (Fig. 3E). Considering that PGC1A is a co-activator of HNF4A and the PPARs^34^, these diet-induced changes in *Ppargc1* expression could amplify the effects of the HFDs on the NRs.

In the jejunum, most of the 24 DEGs were between VIV chow and the three HFDs, with little difference between the HFDs (Fig. 3B). The exception was *Scd1*, which had much higher expression in the CO diet compared to the other HFDs and the VIV chow (Fig. 3F), consistent with the function of SCD1, a desaturase enzyme that introduces double bonds into saturated fatty acids.

The terminal ileum had the fewest HFD-dysregulated genes (18 DEGs) related to barrier function (Fig. 3C). The most dysregulated gene was Resistin-like molecule (RELM) β (*Retnlb*), a cysteine-rich cytokine that plays a role in insulin resistance, gastrointestinal nematode resistance, barrier integrity and susceptibility to inflammation^35^. *Retnlb* expression was decreased by all three HFDs in the terminal ileum (as well as the duodenum) (see Fig. 6B). Since the terminal ileum is the region of the intestines that harbors many bacteria, viruses, and other pathogens, a downregulation in *Retnlb* caused by a HFD could weaken the body’s defenses. One notable gene showing differential expression with HFDs in the proximal colon is the IBD susceptibility gene *Ptpn11* (down in CO vs VIV and SO + CO), which encodes a tyrosine phosphatase involved in the homeostasis of epithelial barrier cells^36^ (Fig. 3G).

### HFD impacts the expression of genes associated with IBD and colon cancer

**Figure 4:**
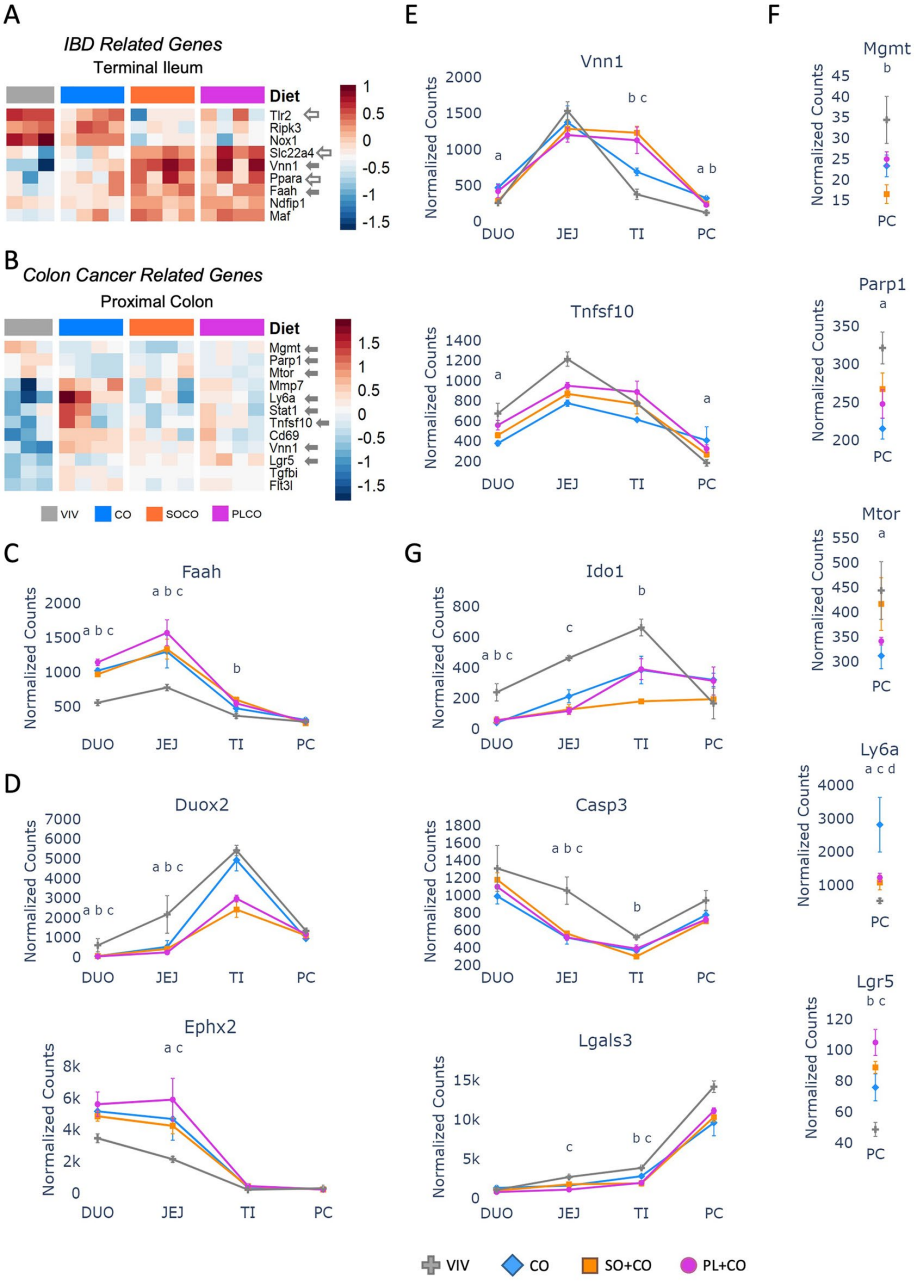
HFDs alter the expression of genes associated with Inflammatory Bowel Disease (IBD) and colon cancer. (**A**,**B**) Heatmaps of genes involved in IBD and colon cancer in the terminal ileum and proximal colon, respectively, of mice fed either low fat VIV chow or one of the three HFDs. Included are genes that are significantly different between any two diets (p-adj ≤ 0.05). N = 3 for Viv and 4 for HFDs per tissue. Solid arrows, plotted in this figure; open arrows, plotted in a subsequent figure. Arbitrary scale of relative expression is shown. See Supplementary Table S6 for a complete list of genes and Supplementary Fig. S6 for additional heatmaps of IBD and colon cancer genes. (**C**–**G**) Line graphs showing normalized read counts with standard deviation (SD) of select genes in various parts of the intestines [only proximal colon is shown in (**F**)] on the indicated diets for IBD and colon cancer. Genes with significantly different levels of expression between the diets within a given tissue (p-adj ≤ 0.05) are indicated as follows: a (VIV vs CO); b (VIV vs SO + CO); c (VIV vs PL + CO); d (CO vs SO + CO).

There was also a large number of genes related to IBD (45 out of 141 genes) and colon cancer (51 out of 192 genes) significantly impacted by the HFDs (Fig. 4A,B and Supplementary Fig. S6). Interestingly, in terms of IBD-related genes, the terminal ileum was impacted the most by the HFDs, consistent with this portion of the gut being frequently inflamed in Crohn’s Disease, a form of IBD (Fig. 4A). *Tlr2* (Toll-like receptor 2), *Ripk3* (receptor interacting serine/threonine kinase 3), and *Nox1* (NADPH oxidase 1) all decreased expression in the SO + CO and PL + CO diets compared to the VIV chow and CO diet. In contrast, *Slc22a4* (a member of the solute carrier family), *Vnn1* (vanin 1), *Faah* (fatty acid amide hydrolase), *Ndfip1* (Nedd4 Family Interacting Protein 2), *Maf* (bZIP transcription factor) showed increased expression in the two soybean oil diets (Fig. 4A,C,E). Noteworthy IBD-related genes in the duodenum and/or jejunum that were affected by the HFDs include *Duox2* (dual oxidase 2), a member of the NADPH oxidase family, which was downregulated by the HFDs, and *Ephx2* (epoxide hydrolase 2), an enzyme that converts fatty acid epoxides to bioactive dihydrodiols which was upregulated by the HFDs (Fig. 4D). No genes related to IBD specific to the colon (i.e., ulcerative colitis) were differentially expressed in the proximal colon.

The HFDs also affected the expression of cancer-related genes in the proximal colon (and other parts of the intestines) including *Vnn1*, a pantetheinase with roles in oxidative stress and inflammation^37^, and *Tnfsf10* (tumor necrosis factor ligand superfamily, member 10) (Fig. 4E). Genes specific to colon cancer and altered only in the proximal colon include DNA repair enzymes *Mgmt* (O-6-Methylguanine-DNA Methyltransferase) and *Parp1* (Poly(ADP-Ribose) Polymerase 1) and *Mtor*, a mediator of response to cellular stress including DNA damage—all were downregulated by one or more HFDs. In contrast, *Ly6a* (Lymphocyte Antigen 6A), which regulates T cell proliferation and is a marker for cancer stem cells^38^, and *Lgr5,* a prominent marker for mitotically active crypt intestinal stem cells involved in the Wnt signaling pathway, were upregulated (Fig. 4F). Finally, there were several genes related to colon cancer that were altered by the HFDs but only in the small intestines. For example, *Ido1* (indoleamine 2,3-dioxygenase 1) is the first and rate-limiting step in tryptophan catabolism and plays a role in antimicrobial and anti-tumor defense, neuropathology and immunoregulation, *Casp3* (caspase 3) is a key executor of apoptosis and *Lgals3* (galectin 3) plays a role in innate immunity and T-cell regulation and exhibits antimicrobial activity against bacteria and fungi. All three were downregulated by the HFDs (Fig. 4G).

### Network analysis reveals an impact of HFDs on the immune system as well as metabolism

**Figure 5:**
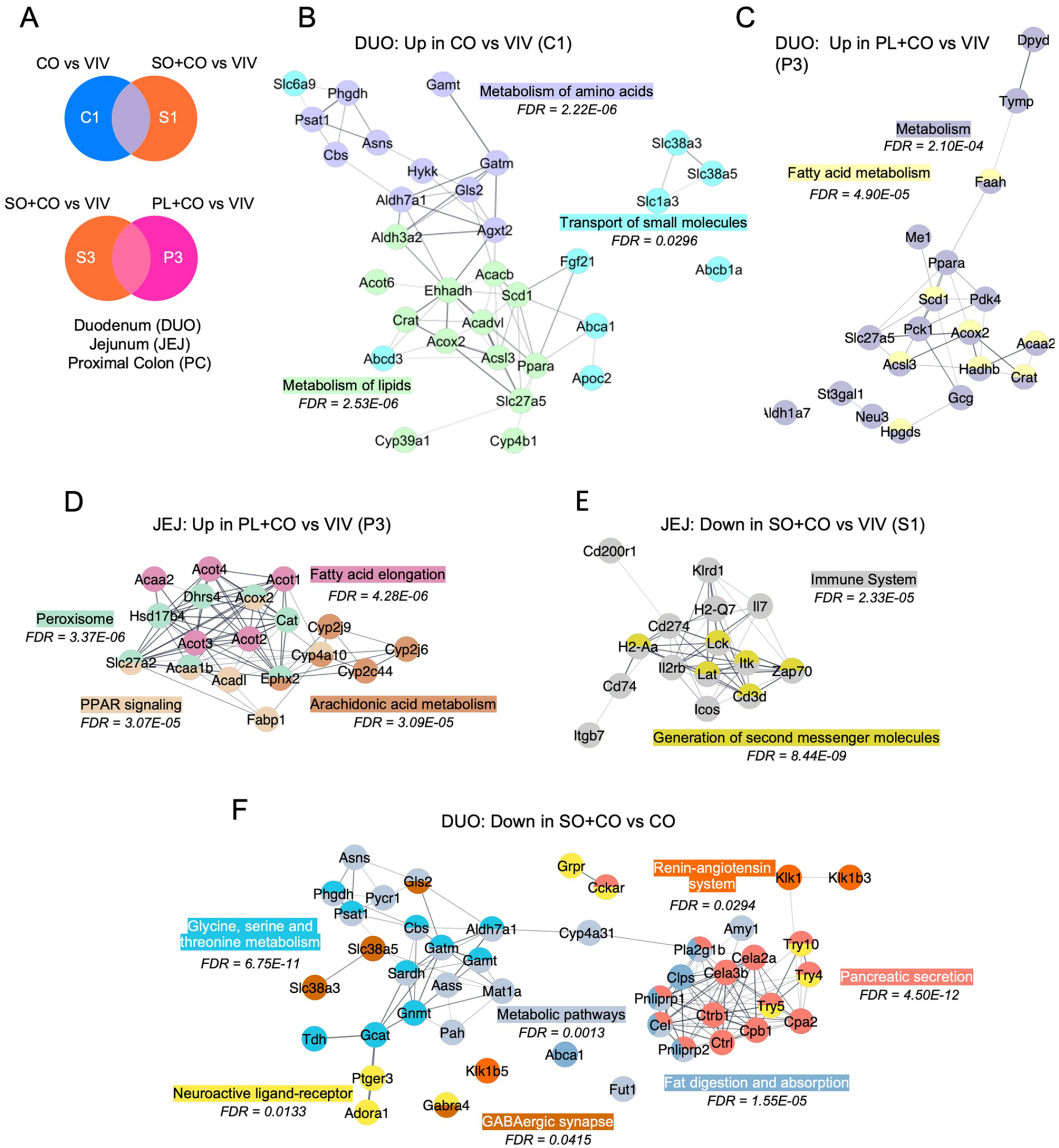
Network analysis of differentially expressed genes (DEGs) in various HFDs and vivarium chow in various parts of the intestines. (**A**) Venn diagram of pairwise comparisons of differentially expressed genes (DEGs) (p-adj ≤ 0.05) between each HFD (CO, SO + CO, PL + CO) and the low-fat Vivarium chow (VIV). (**B**–**F**) Networks of DEGs either up or down-regulated in the various tissues in the indicated portions of the Venn diagram in (**A**). C1: dysregulated in CO vs. VIV but not in SO + CO vs. VIV; S1: dysregulated in SO + CO vs. VIV but not in CO vs. VIV; S3 dysregulated in SO + CO vs. VIV but not in PL + CO vs. VIV; P3 dysregulated in PL + CO vs. VIV but not in SO + CO vs. VIV. Networks were identified in Cytoscape: Reactome (**B**–**E**) or KEGG (**F**). Individual FDRs for the indicated pathways are shown. See Supplementary Fig. S7 for additional networks.

To obtain a more detailed understanding of the pathways impacted by HFDs in the different parts of the intestines, we conducted a Venn analysis of the DEGs in different diet comparisons in each tissue followed by Stringapp in Cytoscape to identify networks of genes, utilizing either the Reactome or the KEGG pathway databases (Fig. 5A, Supplementary Fig. S7A). In the duodenum, genes upregulated in the CO vs. VIV comparison but not in SO + CO were involved in the metabolism of amino acids and lipids, as well in the transport of small molecule pathways (Fig. 5B). Additional metabolic categories, especially involving fatty acids, were identified in the PL + CO vs VIV comparison (Fig. 5C). In contrast, downregulated genes in the duodenum in the CO vs VIV comparison were associated with T cell receptor (TCR) signaling and the innate immune system, while genes down in the SO + CO vs VIV comparison were found in pathways related to pancreatic secretion, chemical carcinogenesis, linoleic acid metabolism and fat digestion and absorption (Supplementary Fig. S7B,C). Similarly, in the jejunum, there were many upregulated genes in specific HFDs vs VIV, including fatty acid elongation, arachidonic acid metabolism and PPAR signaling and peroxisome (PL + CO vs VIV, Fig. 5D) and fatty acid metabolism and Phase I genes (CO vs VIV, Supplementary Fig. S7E). In contrast, as in the duodenum, the downregulated genes in the jejunum were related to the immune system, second messenger molecules, cytokine signaling and herpes simplex infection (SO + CO vs VIV, Fig. 5E, PL + CO vs VIV, Supplementary Fig. S7F,G). In the SO + CO vs CO comparison, the duodenum yielded a completely different mix of downregulated metabolic pathways (including glycine, serine and threonine metabolism), fat digestion and absorption, pancreatic secretion, the renin-angiotensin system (RAS) and, intriguingly, GABAergic synapse and neuroactive ligand-receptor (Fig. 5F) while oxidative phosphorylation genes were upregulated (Supplementary Fig. S7D). Lastly, there was a network of genes down in the proximal colon in the SO + CO vs CO comparison involved in herpes simplex infection, RIG-I-like receptor signaling and cytosolic DNA sensing (Supplementary Fig. S7H). There were no significant networks among the dysregulated genes in the terminal ileum.

### Impact of HFDs on the gut microbiome

**Figure 6:**
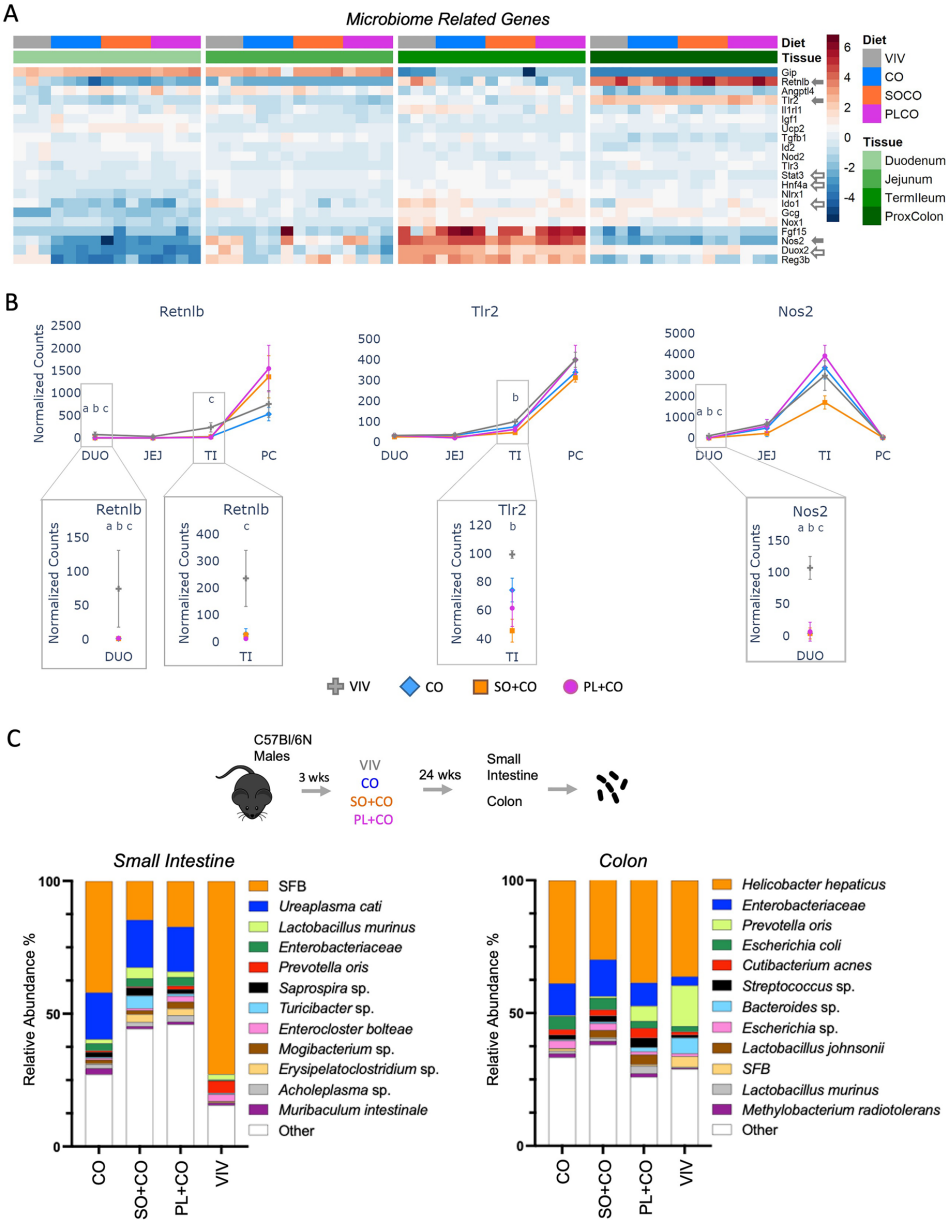
Impact of HFDs on the gut microbiome and related host genes. (**A**) Heatmap of significantly dysregulated genes involved in the microbiome response in the host. Included are genes that are significantly different between any two diets (p-adj ≤ 0.05). Solid arrows, plotted in this figure; open arrows, plotted in a subsequent figure. Arbitrary scale of relative expression is shown. See Supplementary Table S6 for a complete list of genes. (**B**) Line graphs showing normalized read counts with standard deviation (SD) of select genes in various parts of the intestines on the indicated diets. Genes with significantly different levels of expression between the diets within a given tissue (p-adj ≤ 0.05) are indicated as follows: (VIV, CO, SO + CO, PL + CO). a (VIV vs CO); b (VIV vs SO + CO); c (VIV vs PL + CO). (**C**) Taxa plots showing differentially abundant bacteria from host-associated intestinal epithelial cells in the small intestine or colon of mice fed the different diets (CO, SO + CO, PL + CO, VIV). Values in taxa plots are % IlluminaITS rRNA gene reads from intestinal epithelial cells from the indicated tissue. n = 11–12 mice for each of the four diets.

Since HFDs are known to impact the microbiome^39^, we generated a heatmap of 21 microbiome-related genes (out of 99 total) that were significantly dysregulated between any two diets (Fig. 6A). *Retnlb* showed consistently high expression in the proximal colon compared to other tissues and, as noted above, decreased expression by one or more HFD in the terminal ileum as well as the duodenum (Fig. 6B). *Tlr2* (toll like receptor 2), a pattern recognition gene, and *Nos2* (nitric oxide synthase 2), which plays a role in immunity against bacteria, fungi and viruses, were also decreased in one or more HFD in the terminal ileum and duodenum, respectively (Fig. 6B).

Microbiome analysis of the small intestine and colon in the HFDs and VIV chow revealed the presence of many species of bacteria, with their relative abundance influenced by the diet (Fig. 6C). Importantly, there was an increase in populations of various pathogenic and opportunistically pathogenic bacteria in both the small intestines and the colon in the HFDs compared to VIV chow—*Ureaplasma cati*, *Turicibacter* sp. and *Erysipelatoclostridium* sp. in the small intestines and *Enterobacteriaceae* in the colon^40–43^. There was also a notable decrease bacteria typically considered to be beneficial with the HFDs—segmented filamentous bacteria (SFB) in the small intestines and *Bacteroides* and *Prevotella oris* in the colon^44–46^. *Bacteroides* are known to be abundant in the guts of healthy animals on low fat diets but can increase or decrease depending on the type of diet^47,48^ and act as opportunistic pathogens, especially when translocated to other tissues^49^.

### Impact of HFDs on the expression of genes involved in COVID-19

**Figure 7:**
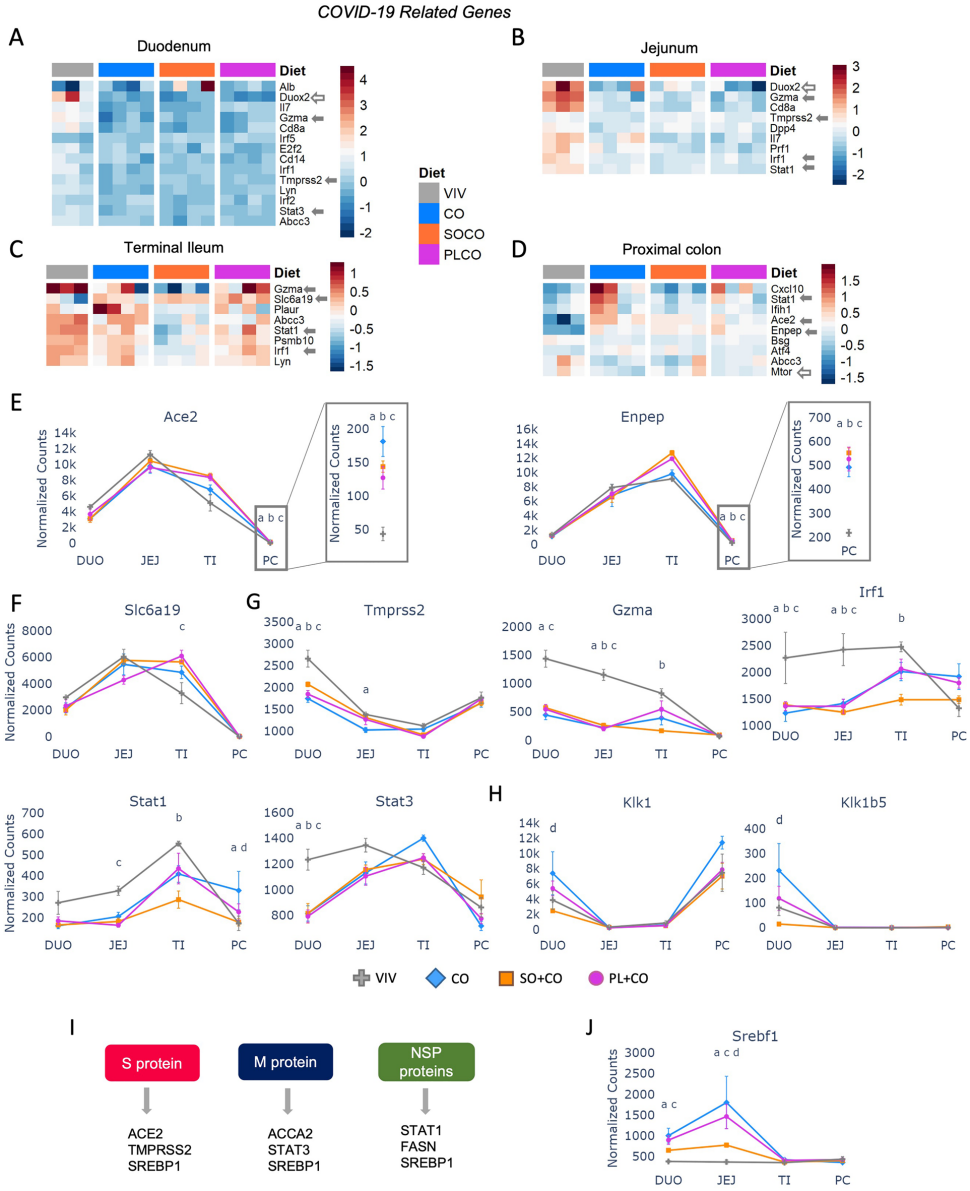
HFDs impact the expression of genes involved in SARS-CoV-2 across the intestinal tract. (**A**–**D**) Heatmaps of significantly dysregulated genes involved in COVID-19. Included are genes that are significantly different between any two diets (p-adj ≤ 0.05). Solid arrows, plotted in this figure; open arrows, plotted in other (main) figures. Arbitrary scale of relative expression is shown. See Supplementary Table S6 for a complete list of genes. (**E**–**H**) Line graphs showing normalized read counts with standard deviation (SD) of select genes in various parts of the intestines on the indicated diets involved in COVID-19. Genes with significantly different levels of expression between the diets within a given tissue (p-adj ≤ 0.05) are indicated as follows: a (VIV vs CO); b (VIV vs SO + CO); c (VIV vs PL + CO); d (CO vs SO + CO). (**I**) Interaction between indicated host proteins and SARS-Co-V2 viral proteins. (**J**) As in (**E**–**H**) but for *Srebf1*.

Although COVID-19 primarily affects the respiratory system, it can also impact the intestinal tract, leading to diarrhea, inflammation and septic shock^50^. Furthermore, patients with COVID-19-related diarrhea are more likely to require hospitalization and experience a more severe infection^50^. Heatmaps revealed several COVID-19-related genes (38 out of 159 total) that were dysregulated by one or more of the HFDs (Fig. 7A–D), including *Ace2* (angiotensin-converting enzyme 2) and *Enpep* (glutamyl aminopeptidase) (Fig. 7E). In the proximal colon, both genes exhibited a significant increase in expression in HFDs compared to VIV chow. *Slc6a19* (solute carrier family 6 member 19) showed increased expression in the terminal ileum in PL + CO vs. VIV chow (Fig. 7F). In contrast, *Tmprss2* (transmembrane Serine protease 2), *Gzma* (granzyme A), *Irf1* (interferon regulatory factor 1), *Stat1* and *Stat3* (signal transducer and activator of transcription 1/3) displayed decreased expression in one or more HFDs compared to VIV chow in various sections of the intestines (Fig. 7G). Moreover, two COVID-19-related genes, *Klk1* and *Klk1b5*, identified in the Renin-angiotensin system (RAS) in the network analysis (Fig. 5F), were upregulated by the CO diet in the duodenum (Fig. 7H). Kallikreins are serum serine proteases that play an important role in the vascular system and have been proposed as therapeutic targets for COVID-19^51,52^.

To further investigate the impact of HFDs on intestinal health during COVID-19, we utilized the BioGRID database^53^ to identify interactions between host proteins dysregulated by the HFDs and viral proteins of SARS-CoV-2, the causative agent of COVID-19. These interactions involved ACE2, TMPRSS2, SREBPF1 with the viral S protein; ACCA2, STAT3, and SREBPF1 with the viral M protein; and STAT1, FASN, and SREBPF1 with the viral NSP proteins (Fig. 7I). Intriguingly, the expression of *Srebf1* (sterol regulatory element binding transcription factor 1), was significantly increased in the duodenum and jejunum in response to HFDs, which could promote increased interaction between this host protein and the viral NSP protein (Fig. 7J).

## Discussion

To our knowledge, this is the first comprehensive RNA-seq analysis conducted in four different sections of the intestines (duodenum, jejunum, terminal ileum and proximal colon) and comparing three distinct HFDs to a standard low-fat diet. The HFDs used here are formulated with an amount of fat closer to that consumed by Americans (40% kcal) and the most prevalent cooking oil used in the United States, soybean oil (SO) which is high in LA, a genetically modified soybean oil Plenish (PL) low in LA but high in oleic acid, and coconut oil (CO) consisting primarily of saturated fats lauric acid (C12:0) and myristic acid (C14:0). Importantly, the SO + CO diet resulted in a greater number of dysregulated genes compared to the CO diet than did the PL + CO diet, suggesting that excess LA has a greater impact on intestinal gene expression than oleic acid (Fig. 1). These results are consistent with differential effects of SO and Plenish we have observed previously in terms of obesity, diabetes and colitis^3,4^.

**Figure 8:**
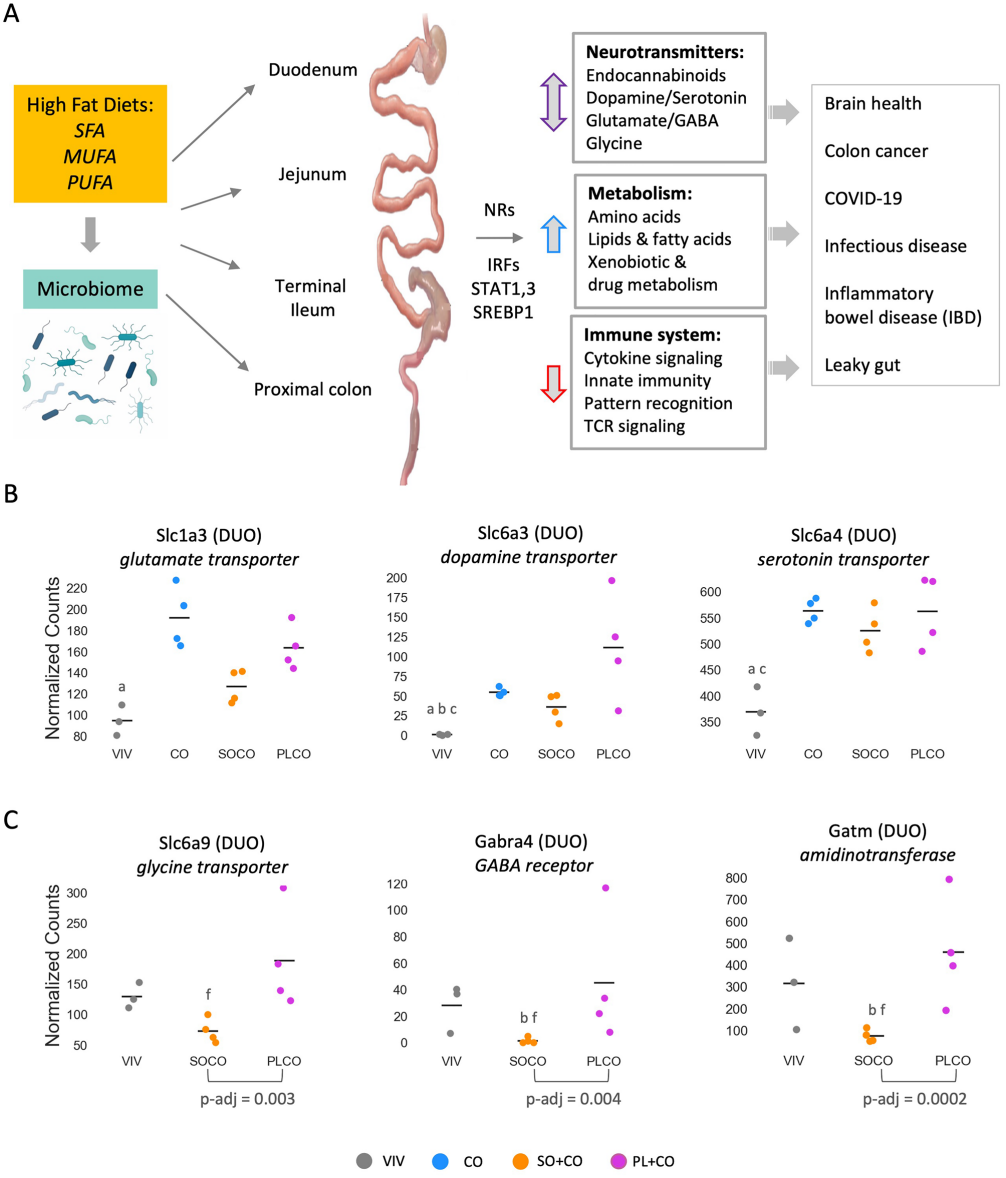
Overview of impact of HFDs on microbiome and gene expression. (**A**) Overview of the role various HFDs may play in the development of disease by impacting the indicated pathways along the intestinal tract. *SFA* saturated fatty acids, *MUFA* monounsaturated fatty acids, *PUFA* polyunsaturated fatty acids. Image for microbiome obtained from Biorender.com. See “Discussion” for details. (**B**) Scatter plots showing normalized read counts of select intestinal transporters in various parts of the intestines on the indicated diets (VIV, CO, SO + CO, PL + CO). Line, mean of biological replicates. Genes with significantly different levels of expression between the diets within a given tissue (p-adj ≤ 0.02) are indicated as follows: a (VIV vs CO); b (VIV vs SO + CO); c (VIV vs PL + CO); f (SO + CO vs PL + CO). (**C**) As in B but for VIV, SO + CO and PL + CO diets. p-adj between SO + CO and PL + CO diets is indicated. Values for CO were higher than the other diets but had a very wide range and hence not plotted: see Supplementary Table S2 for numerical values.

The majority of dysregulated genes can be grouped into one of two categories—metabolism (generally increased) and the immune system (typically decreased)—and are associated with various pathological conditions and diseases ranging from colon cancer, inflammation and IBD to leaky gut and infectious diseases including COVID-19. There were also several genes involved in the metabolism or transport of neurotransmitters—including endocannabinoids, dopamine and serotonin, gamma-aminobutyric acid (GABA), glutamate and glycine—that are dysregulated by the HFDs and could impact brain health. Lastly, we observed changes in a number of transcriptional regulators—including NRs, IRFs, STATs and SREBP1—that could play a role in regulating the expression of the genes in the other categories (Fig. 8A). Taken together, our findings are consistent with the notion that the gut-microbiome-brain axis may be influenced by what we eat and affect not only metabolism and the immune function but also brain health^54^.

### HFDs impact expression of intestinal genes involved in fatty acid and drug metabolism

Perhaps the best example of a gene involved in fatty acid metabolism that is impacted by diet is *Scd1* which converts saturated fatty acids to monounsaturated fatty acids; it is upregulated by CO more than tenfold in the jejunum (Fig. 3). Other genes include those that impact LA and its downstream metabolite arachidonic acid which is associated with pro-inflammatory processes (e.g., *Cyp2c, Cyp2j, Cyp4a*, *Ephx2*) (Fig. 5, Supplementary Fig. S7). Consistent with the increased expression of *Ephx2* in the HFDs, we recently showed that a diet high in SO leads to increased levels of oxylipins in the intestines and correlates with barrier dysfunction and susceptibility to colitis in mice^4^. Changes in genes involved in amino acid metabolism (Fig. 5) were less anticipated given that the diets all contained the same amount of protein but an intriguing finding nonetheless as they could play a role in select signaling pathways.

Dysregulation of numerous genes involved in xenobiotic and drug metabolism (Supplementary Figs. S2, S3, S4) is consistent with the notion that diet impacts Phase I and Phase II reactions in the liver^55^, although to our knowledge this is the first report of different cooking oils impacting *Cyp, Gst* and *Ugt* gene expression in different parts of the intestines. *Cyp2d26*, for example, is upregulated by more than one HFD: its human ortholog, *CYP2D6*, is known to metabolize numerous drugs including antidepressants, antipsychotics, analgesics, antitussives, beta-adrenergic blocking agents, antiarrhythmics, and antiemetics^28^ (Fig. 1). These results suggest that the intestines may play a more significant role in drug metabolism than previously recognized and that there could be important health consequences if a basic component of one’s diet such as cooking oil is changed.

### HFDs impact expression of intestinal genes involved in the immune system, the microbiome and neurological signaling

Given that the intestine is the first line of defense against many foreign invaders and plays a critical role in immune function, it is notable that we identified many genes linked to the immune system that were downregulated by one or more HFD. For example, we observed dysregulation of genes involved in innate immunity (e.g., *Retnlb*, *Reg3b*), cytokine signaling (e.g., *Ccl8*, *Ccl20*, *Ccl22*, *Tnfsf10*), and pattern recognition (e.g., *Tlr2*, *Tlr3*) in response to the different HFDs, even without exposure to an external pathogenic agent. *Retnlb* and *Reg3b* both have antibacterial properties and *Reg3b* is regulated by *Retnlb*^35,56–58^ (Figs. 4, 5, 6).

While the effects of diet on the microbiome are well established, especially in terms of fiber and polyphenols^54,59^, less well studied are the effects of different dietary fatty acids. We observed in all three HFDs an increase in *Enterobacteriaceae* in the small intestine, a group of organisms known to enhance the inflammatory response^60^. In contrast, there was an increase in *Turicibacter*, a bacterium associated with increased adiposity^61^ but only in SO + CO, consistent with that diet causing more obesity than either CO or PL + CO^3^. Further investigation is required to determine whether changes in the microbiome are a direct result of the diets or, alternatively, are a result of changes in the host immune system.

Host genes implicated in the tryptophan-serotonin pathway, which is known to be impacted by the gut microbiota, were also dysregulated by the HFDs. For example, *Ido1*, which encodes an enzyme that generates the neurotransmitter serotonin, was downregulated by the HFDs (Fig. 4). In contrast, several neurotransmitter transporters were upregulated by one or more HFD—glutamate transporter *Slc1a3*, dopamine transporter *Slc6a3*, serotonin transporter *Slc6a4* (Fig. 8B) and amino acid transporters *Slc38a3* and *Slc38a5* (Fig. 5). While these transporters are known to be associated with various addictions (e.g., alcohol, cocaine, nicotine), behaviors (e.g., attention-deficit hyperactivity disorder, gambling) and diseases (e.g., neuroinflammation, epilepsy, Parkinson’s, depression, ataxia), additional studies are required to determine the net physiological effect, if any, of the dysregulation of these genes by the HFDs^28,62^.

*Faah,* which was greatly upregulated by all three HFDs (Fig. 4), is a hydrolase for endocannabinoids and N-acylethanolamines such as 2-arachidonoylglycerol (2-AG) and *N*-arachidonoylethanolamine (AEA)^63^. This suggests that the HFDs might cause decreased levels of endocannabinoids in the gut which is what we observed with a soybean oil diet^4^. Although only a total of 55 genes were dysregulated between the SO + CO and PL + CO diets, several of the high LA soybean oil-specific genes were involved in neurotransmitter signaling—e.g., glycine transporter *Slc6a9,* GABA receptor *Gabra4*, and *Gatm,* an amidinotransferase involved in creatine biosynthesis critical for proper cognition, language and behavior. All were significantly downregulated in SO + CO compared to low LA/high oleic acid diet (PL + CO) (Supplementary Tables S2–S5, Fig. 8C). On a related note, we have previously reported that the same diets used in this study also impact the transcriptome of the hypothalamus and many of those genes are related to mental health^64^.

### HFDs impact expression of genes involved in transcription regulation

One potential mechanism by which different dietary fats could alter the expression of so many genes in the intestines is via nuclear receptors (NRs) which respond to hydrophobic ligands, including fatty acids. While assessing the impact of the dietary fats on the transcriptional activity of NRs in the gut is beyond the scope of this study, we did observe changes in expression of two NRs that bind fatty acids—PPARα and HNF4α—as well as NR co-regulators such as SHP (*Nr0b2*) and PGC1A (*Ppargc1a*) (Fig. 2). HNF4﻿α, downregulated by PL + CO in the duodenum, binds LA and plays a critical role in maintaining intestinal health, intestinal epithelial differentiation and barrier function^65–67^; it is also dysregulated in colon cancer as well as colitis and is an IBD susceptibility gene^4,68,69^. *Retnlb*, downregulated by HFD in the duodenum, is a known HNF4﻿α target gene^65^. In contrast, PPAR﻿α, which is known to play a protective role against colon cancer^70,71^, was upregulated by all three HFDs in the small intestines, as was *Cd36*, a long chain fatty acid transporter and target of PPAR﻿α^72^. The other most prominent transcription factor family dysregulated by the HFDs were the STAT/IRF factors involved in interferon signaling (*Stat1*, *Stat3*, *Irf1*, *Irf5*, *Irf8*); their downregulation in the HFDs could suggest a potentially compromised immune system. Lastly, SREBPF1, which regulates the expression of fatty acid and cholesterol metabolism genes, including *Scd1*, is upregulated by the HFDs and is potentially linked to COVID-19 (Fig. 7)^73^.

### Impact of HFDs on genes involved in barrier function, IBD and colon cancer

We observed changes in expression of many genes by one or more HFDs which could contribute to intestinal disease (Figs. 3, 4). There was a decrease in expression of a number of anti-cancer genes (e.g., *Casp3*, *Mgmt*, *Parp1*, *Ptpn11*) as well as an increase in several cancer-promoting genes (e.g., *Ly6a*, *Lgr5*, *Vnn1*). There were also a couple examples where the HFDs seemed to be protective: decreased expression of *Duox2* in HFDs suggests a lower inflammatory response compared to the control diet^74^, which could be beneficial in halting the progression of colorectal cancer^75^, while reduced expression of *Ripk3* in the terminal ileum may help alleviate inflammation in IBD^76^. It is possible that some of these beneficial changes in gene expression could be due to a physiological response of the body to fight inflammation caused by the HFD. Some genes showed differential effects depending on the HFD. For example, *Cldn10* and *EGF* have lower expression in SO + CO vs PL + CO: reductions in both of these genes can impair barrier function, consistent with our previous report of a high LA diet contributing to barrier dysfunction while olive oil, a key feature of the Mediterranean diet, is anti-inflammatory^4,77,78^.

### Effects of HFDs on COVID-19-related genes

Obesity is a significant risk factor for COVID-19 and COVID-19 patients can experience gastrointestinal symptoms, including damage to the intestinal epithelial barrier^79,80^. Therefore, it is perhaps not surprising that the lower gastrointestinal tract has a large number of ACE2 receptors and that its expression, along with genes that encode ENPEP and SLC6A19 which facilitate viral entry via ACE2^81,82^, is increased in one or more of the HFDs (Fig. 7). Like ACE2, *Klk1* and *Klk1b5* are part of the RAS pathway and are thought to be required for viral processing^83^; their expression was also increased in the CO diet. These results, along with the downregulation of several host genes involved in the immune response to SARS-CoV-2 (*Gzma*, *Irf1*, *Stat1*, *Stat3*), suggest that HFDs might be detrimental to COVID-19 patients (Fig. 7).

### Limitations and caveats

Given the length of time on the diets (24 weeks), the observed changes in gene expression could be due directly to the diets and/or to their long-term effects such as obesity, diabetes and susceptibility to colitis^2–4^. Even though all three HFDs lacked fiber, they often displayed different effects on gene expression suggesting that not all of the observed effects are due to a lack of fiber. Whole tissue was used so in addition to intestinal epithelial cells other cell types, including immune cells, would have been sampled: single-cell RNAseq shows that a HFD does indeed impact different cell types in a differential fashion, and differences can be observed within days^12^. Finally, the relevance to humans must be established. Since most of the DEGs highlighted in the study are highly conserved between mouse and human, including several of the transcriptional regulators—HNF4﻿α, PPAR﻿α, STAT1/3, IRF1, SREBPF1 are all over 80% identical between human and mouse on the protein level—we anticipate that many of the effects reported here will also be found in humans.

### Supplementary Information

Supplementary Figures.

Supplementary Table S1.

Supplementary Table S2.

Supplementary Table S3.

Supplementary Table S4.

Supplementary Table S5.

Supplementary Table S6.

## Data Availability

All data generated or analyzed during this study are included in this article and its Supplementary Information Files. The raw RNA-seq data are publicly available in Gene Expression Omnibus (GEO), accession number GSE220302. DNA sequencing data of the microbiome is publicly available at SRA BioProject, Accession #PRJNA615924.
