# Supplementary material for: Impact of various high fat diets on gene expression and the microbiome across the mouse intestines
Source: Sci Rep. 2023 Dec 27;13:22758. doi: 10.1038/s41598-023-49555-7 (PMC10752901; doi:10.1038/s41598-023-49555-7)
Supplement: Supplementary file 1 — Supplementary Figures. [file 41598_2023_49555_MOESM1_ESM.pdf]

Supplementary Information

Impact of Various High Fat Diets on Gene Expression and the Microbiome Across the Mouse Intestines

Martinez-Lomeli, Deol et al., 2023

This files contains Supplementary Figures S1-S7.

Published as separate files:

Supplementary Table S1: Composition of diets

Supplementary Table S2: Duodenum RNA-seq

Supplementary Table S3: Jejunum RNA-seq

Supplementary Table S4: Terminal ileum RNA-seq

Supplementary Table S5: Proximal colon RNA-seq

Supplementary Table S6: Gene lists for heatmaps

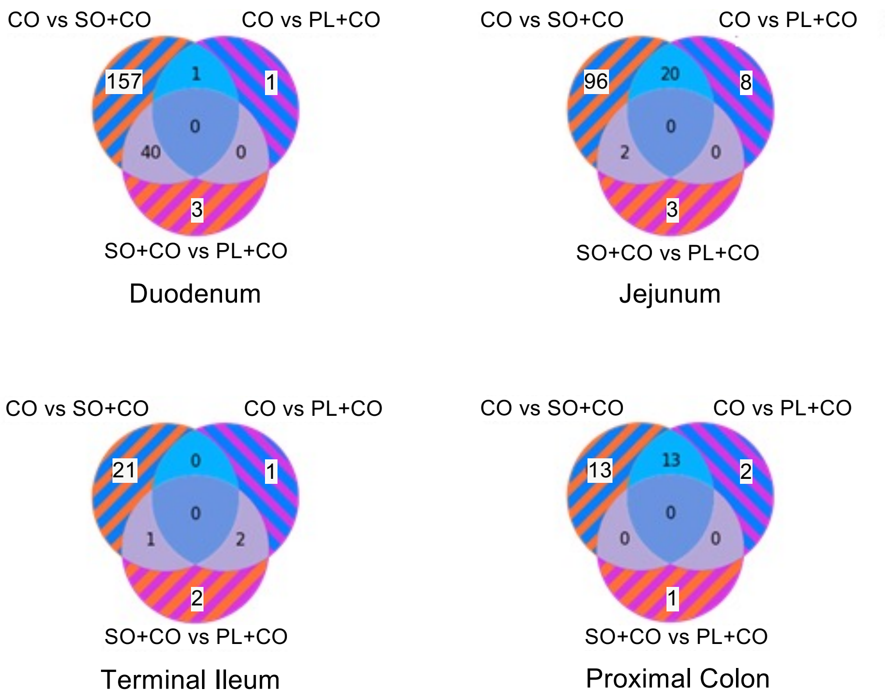

Supplementary Figure S1. Venn diagrams showing the overlap of the DEGs (padj <0.05, abs(Log2FC>1)) in the indicated diet comparisons across the tissues of the intestines.

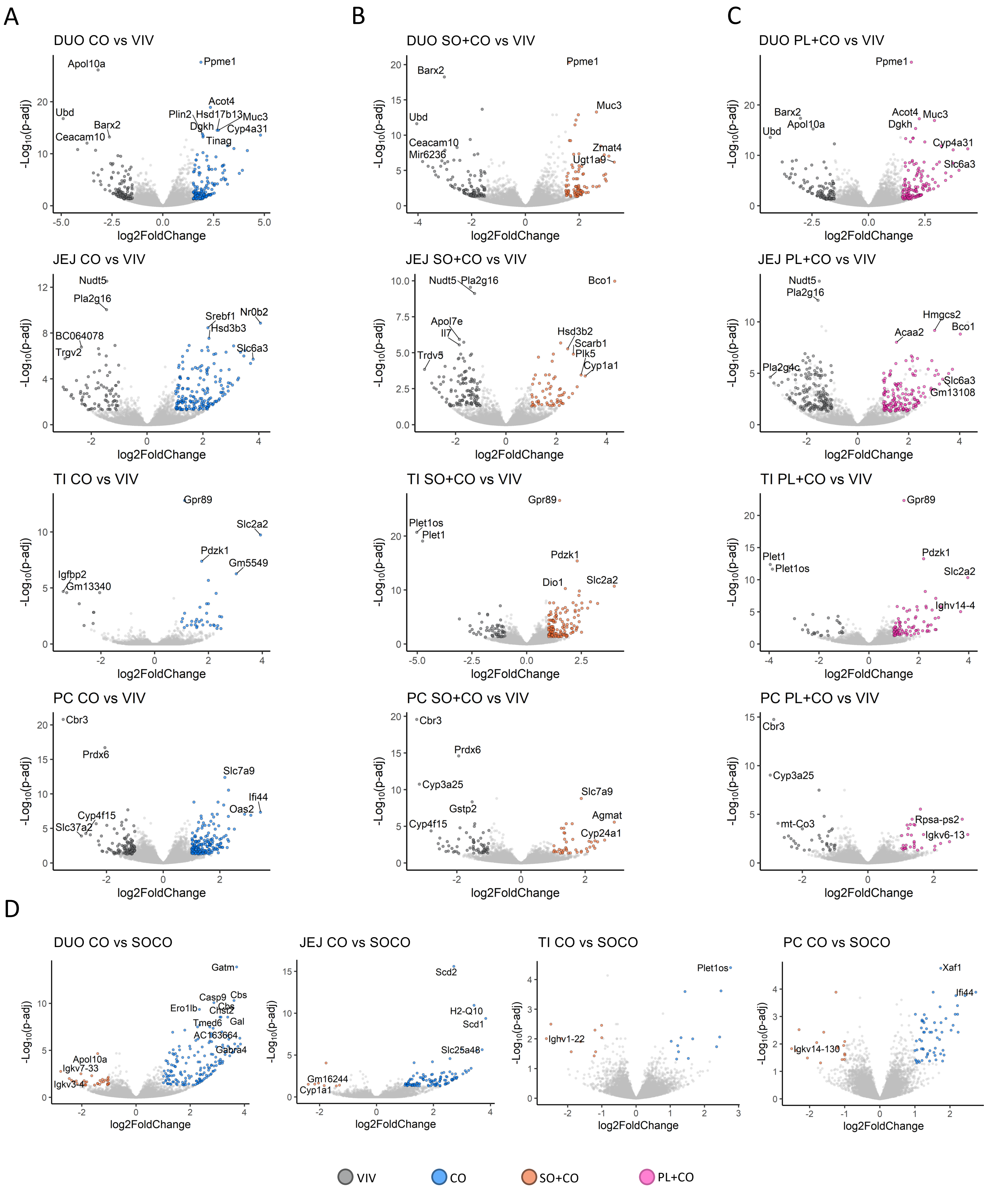

A
B
C
D
DUO CO vs VIV
DUO SO+CO vs VIV
DUO PL+CO vs VIV
JEJ CO vs VIV
JEJ SO+CO vs VIV
JEJ PL+CO vs VIV
TI CO vs VIV
TI SO+CO vs VIV
TI PL+CO vs VIV
PC CO vs VIV
PC SO+CO vs VIV
PC PL+CO vs VIV
DUO CO vs SOCO
JEJ CO vs SOCO
TI CO vs SOCO
PC CO vs SOCO
-Log10(p-adj)
log2FoldChange
VIV
CO
SO+CO
PL+CO

Supplementary Figure S2. Volcano plots of differentially expressed genes (DEGs) in different high fat diets (HFDs) and across different intestinal tissues in mouse.

Plots showing DEGs in duodenum (DUO), jejunum (JEJ), terminal ileum (TI) and proximal colon (PC) in CO vs VIV (**A**), SO+CO vs VIV (**B**) and PL+CO vs VIV (**C**) diets. Plots showing DEGs in CO vs SO+CO diet in different tissues (**D**). Colored spots are genes up or down with p-adj < 0.05 and abs(Log2FC) >0.05. Spots with gene symbols are in the top 5% of significance (-Log10(p-adj)) and have an abs(Log2FC) > 1.5 in the given comparison. Not shown, pairwise comparison between CO vs PLCO since the the number of DEGs was less than 30.

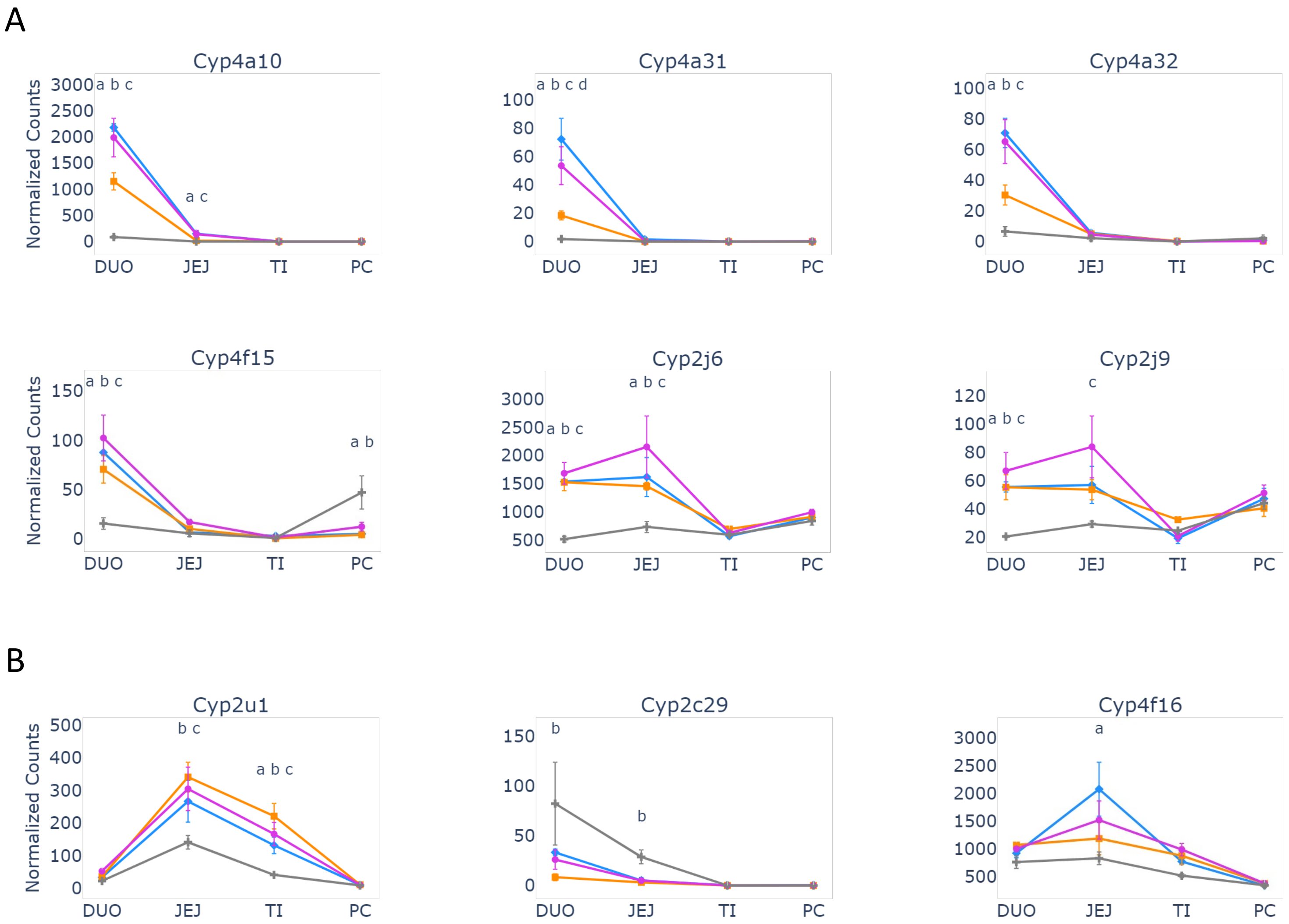

Supplementary Figure S3. HFD impacts the expression of *Cyp* genes in the mouse intestines. Line graphs of average normalized read counts with standard deviation of select *Cyp* genes on the indicated diets (VIV, CO, SO+CO, PL+CO). **A.** Duodenum. **B.** Jejunum. Genes with significantly different levels of expression between the diets within a given tissue (p-adj < 0.05 & abs(Log2FC) >1) are indicated as follows: a (VIV vs Co); b (VIV vs SO+CO); c (VIV vs PL+CO); d (CO vs SO+CO); e (CO vs PL+CO); f (SO+CO vs PL+CO).

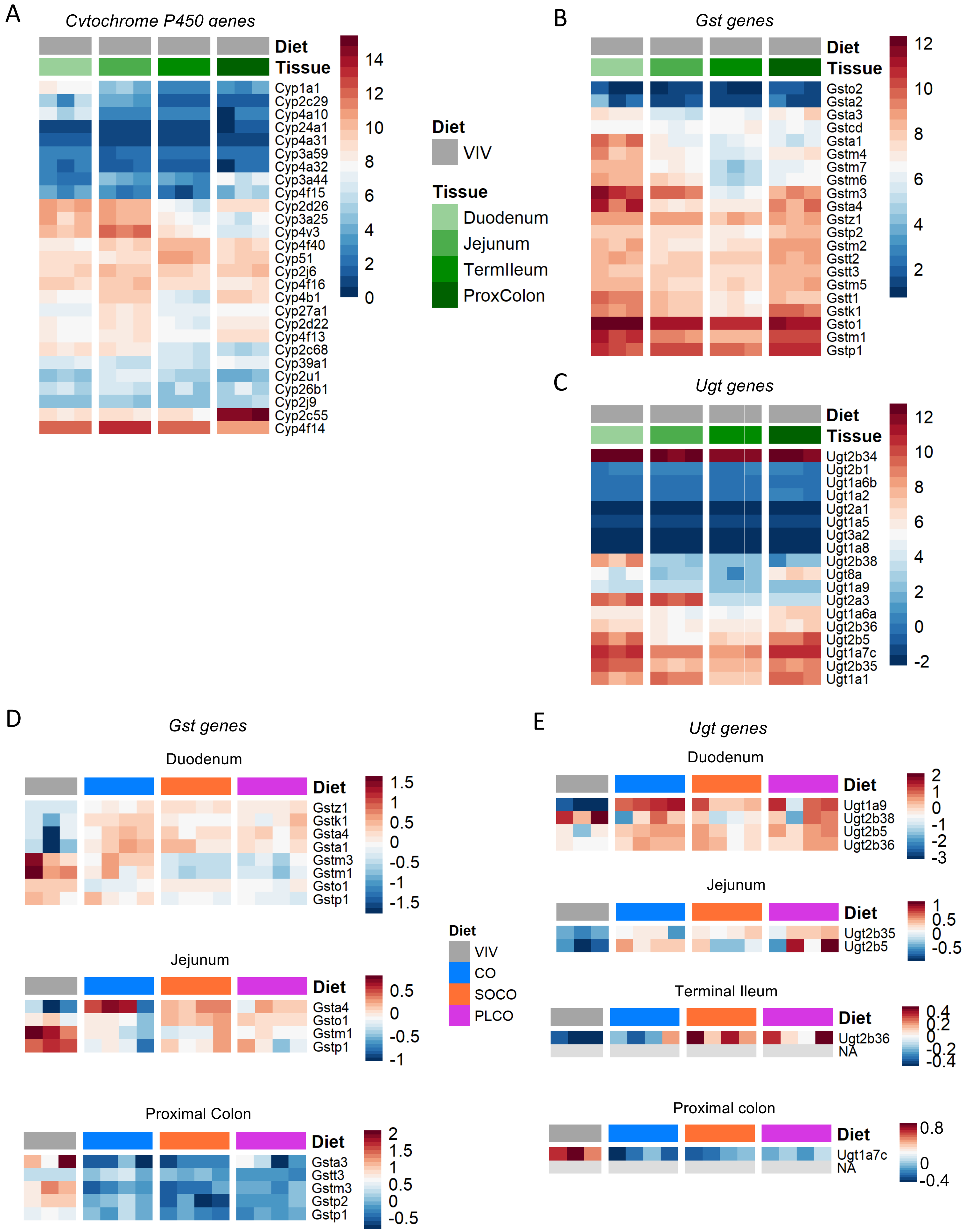
A
Cytochrome P450 genes
Diet
Tissue
Cyp1a1
Cyp2c29
Cyp4a10
Cyp24a1
Cyp4a31
Cyp3a59
Cyp4a32
Cyp3a44
Cyp4f15
Cyp2d26
Cyp3a25
Cyp4v3
Cyp4f40
Cyp51
Cyp2j6
Cyp4f16
Cyp4b1
Cyp27a1
Cyp2d22
Cyp4f13
Cyp2c68
Cyp39a1
Cyp2u1
Cyp26b1
Cyp2j9
Cyp2c55
Cyp4f14
14
12
10
8
6
4
2
0
Diet
VIV
Tissue
Duodenum
Jejunum
TermIleum
ProxColon
B
Gst genes
Diet
Tissue
Gsto2
Gsta2
Gsta3
Gstcd
Gsta1
Gstm4
Gstm7
Gstm6
Gstm3
Gsta4
Gstz1
Gstp2
Gstm2
Gstt2
Gstt3
Gstm5
Gstt1
Gstk1
Gsto1
Gstm1
Gstp1
12
10
8
6
4
2
C
Ugt genes
Diet
Tissue
Ugt2b34
Ugt2b1
Ugt1a6b
Ugt1a2
Ugt2a1
Ugt1a5
Ugt3a2
Ugt1a8
Ugt2b38
Ugt8a
Ugt1a9
Ugt2a3
Ugt1a6a
Ugt2b36
Ugt2b5
Ugt1a7c
Ugt2b35
Ugt1a1
12
10
8
6
4
2
0
-2
D
Gst genes
Duodenum
Diet
Gstz1
Gstk1
Gsta4
Gsta1
Gstm3
Gstm1
Gsto1
Gstp1
1.5
1
0.5
0
-0.5
-1
-1.5
Jejunum
Diet
Gsta4
Gsto1
Gstm1
Gstp1
0.5
0
-0.5
-1
Proximal Colon
Diet
Gsta3
Gstt3
Gstm3
Gstp2
Gstp1
2
1.5
1
0.5
0
-0.5
Diet
VIV
CO
SOCO
PLCO
E
Ugt genes
Duodenum
Diet
Ugt1a9
Ugt2b38
Ugt2b5
Ugt2b36
2
1
0
-1
-2
-3
Jejunum
Diet
Ugt2b35
Ugt2b5
1
0.5
0
-0.5
Terminal Ileum
Diet
Ugt2b36
NA
0.4
0.2
0
-0.2
-0.4
Proximal colon
Diet
Ugt1a7c
NA
0.8
0
-0.4

F

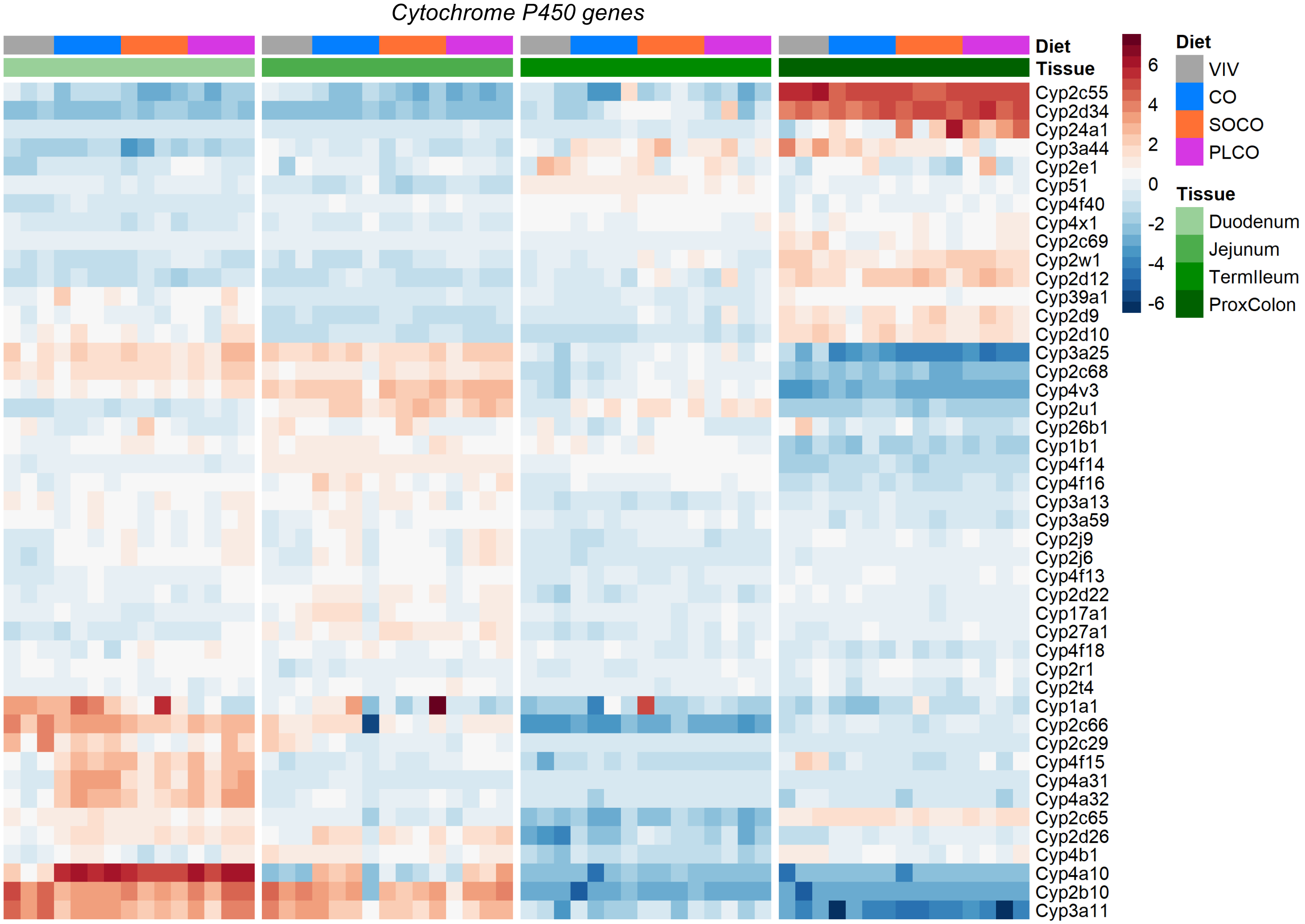

Supplementary Figure S4. Expression of *Cyp*, *Gst* and *Ugt* genes across the intestines of mice fed Vivarium chow and three HFDs.
Non row-normalized heatmaps showing levels of expression of *Cyp* (**A**), *Gst* (**B**) and *Ugt* (**C**) genes in mice fed a low-fat vivarium chow (VIV) diet. Included are genes that are significantly different between any two diets (VIV, CO, SO+CO, PL+CO) (p-adj $\leq$ 0.05) for *Cyp* genes. All the *Gst* and *Ugt* genes were included, regardless of whether there was a significant difference between any diets. **D, E**. Row-normalized heatmaps of *Gst* genes (**D**) and Ugt genes (**E**) with a significant difference (padj<0.05) between any two diets in the indicated tissues. There were no *Gst* genes with a significant difference between diets in the terminal ileum. NA, row automatically added by the pheatmap package in R so that there is more than one gene. **F**. Row normalized heatmap of the *Cyp* genes across four tissues and four diets with a significant difference (padj<0.05) between any two diets. All panels, arbitrary scale of relative expression is shown.

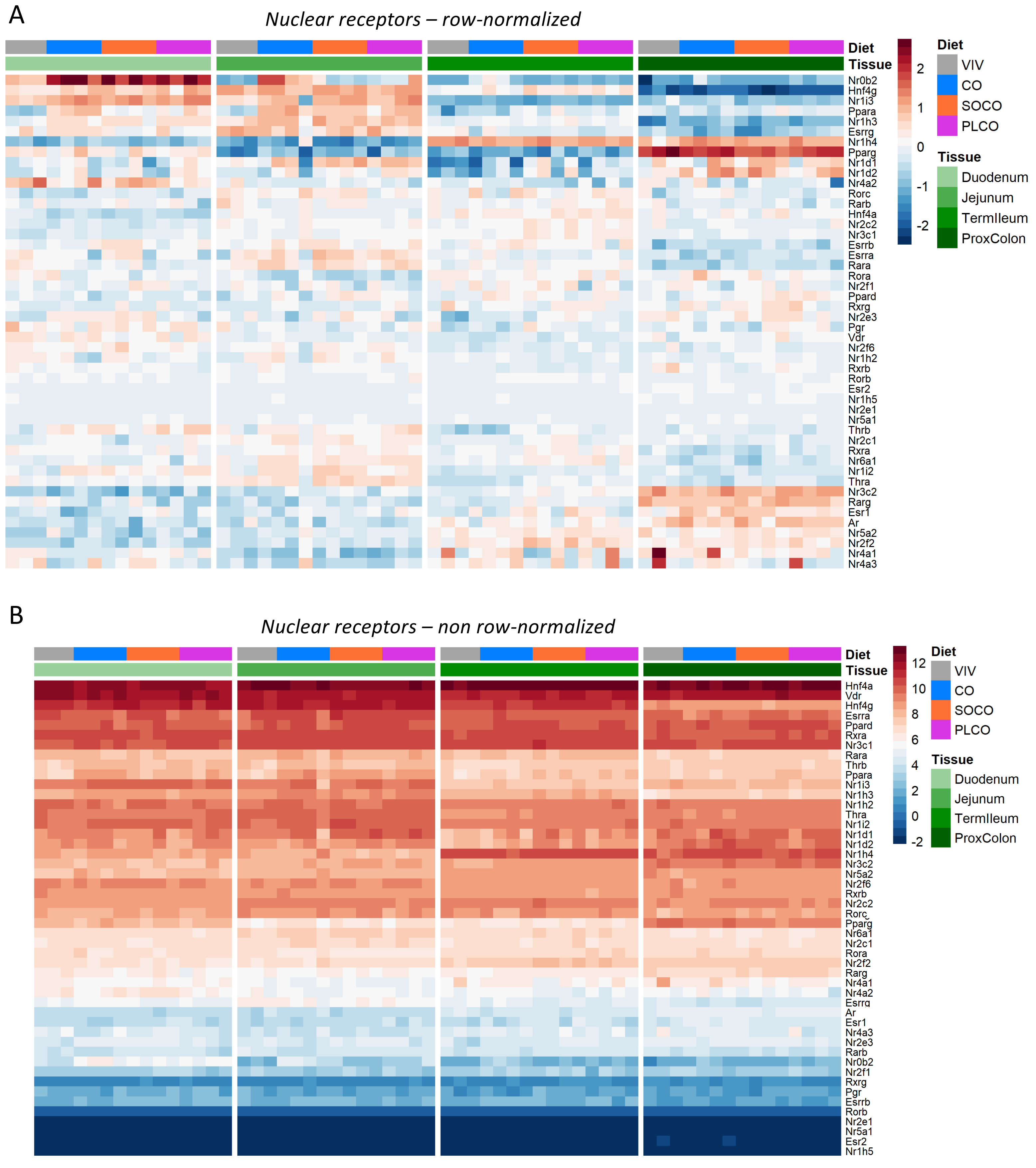

Supplementary Figure S5. Differential expression of nuclear receptor genes across the intestinal tract and different diets in mouse.
Heatmaps showing all the nuclear receptor (NR) genes in mice across all tissues and three HFDs (CO, SOCO, PLCO) and VIV chow, sorted by levels in the duodenum. Normalized read counts across three to four biological replicates are shown across the four different tissues. **A**. Row-normalized heatmap. **B**. Non row-normalized heatmap.

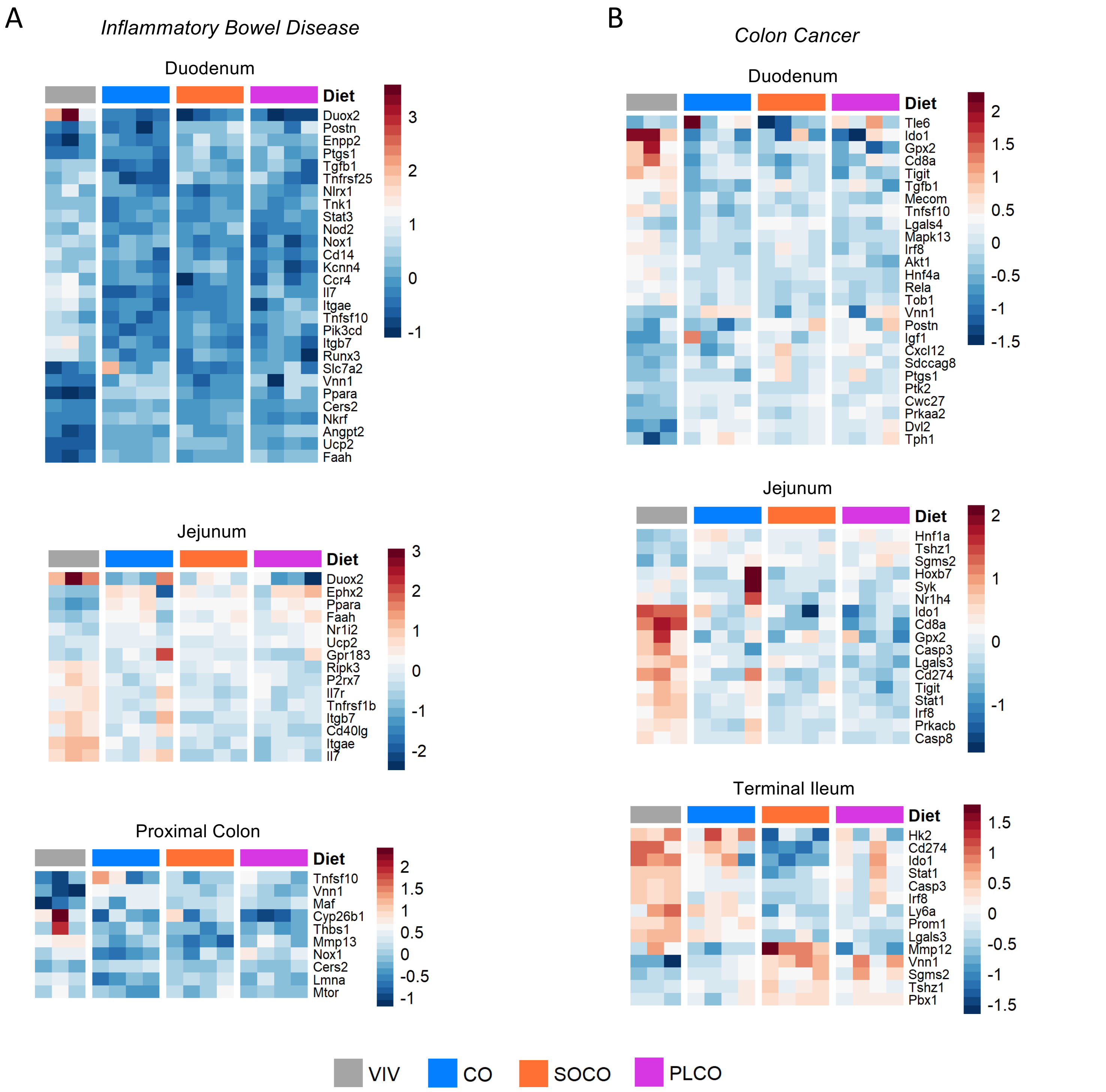

Supplementary Figure S6. HFDs alter the expression of genes associated with Inflammatory Bowel Disease (IBD) and colon cancer in the mouse intestines.
Row-normalized heatmaps of genes involved in IBD (**A**) and colon cancer (**B**) in the indicated tissues of mice fed either low fat VIV chow or one of the three HFDs (CO, SOCO, PLCO). Included are genes that are significantly different between any two diets (p-adj < 0.05). N= 3 for Viv and N=4 for each HFD. Arbitrary scale of relative expression is shown.

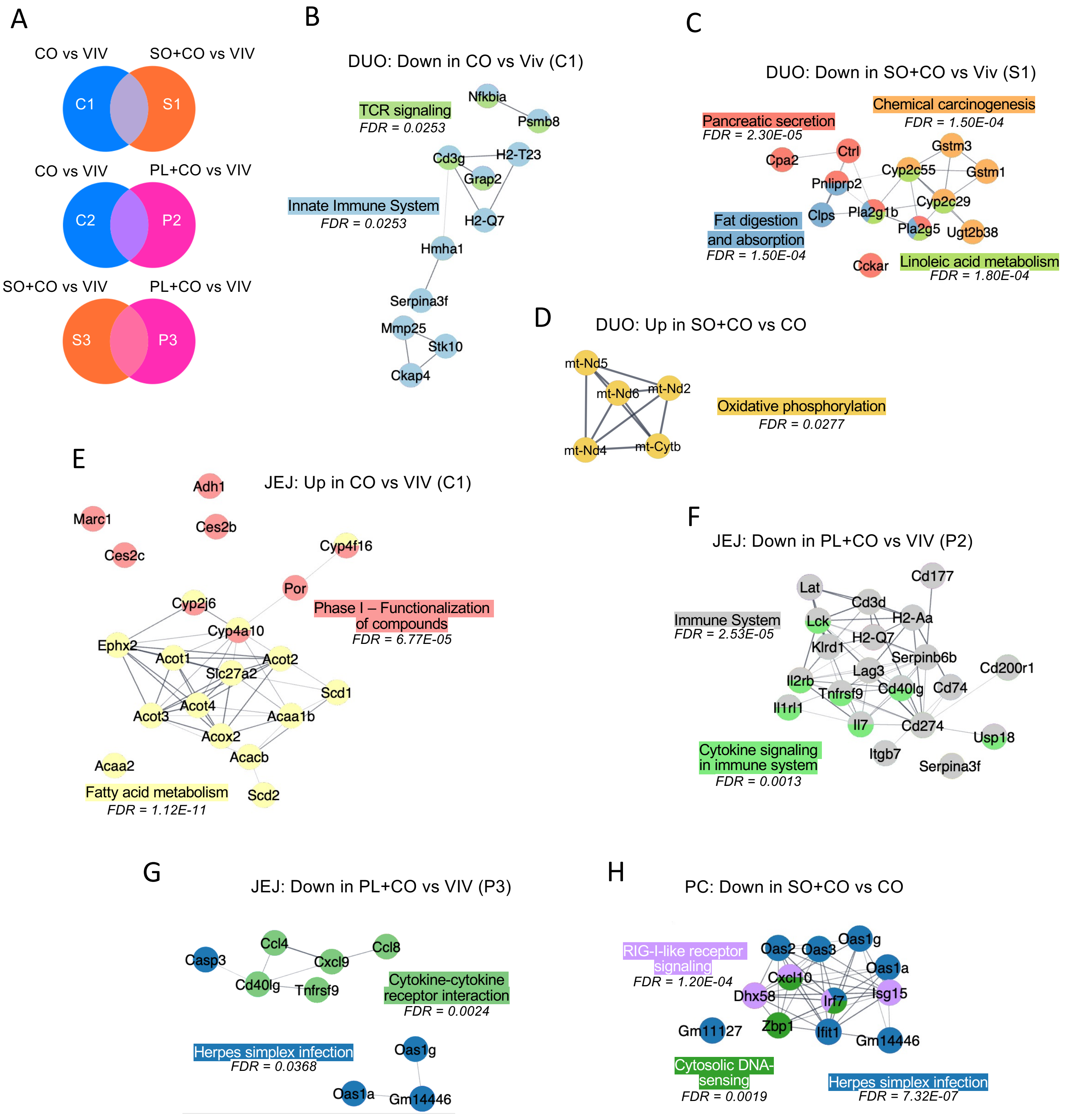

Supplementary Figure S7. Network analysis of differentially expressed genes in various HFDs and VIV chow in the duodenum, jejunum and proximal colon.

A. Venn diagram of pairwise comparisons of differentially expressed genes (DEGs) (p-adj $\leq$ 0.05) between HFDs (CO, SO+CO, PL+CO) and the low-fat Vivarium chow (VIV). **B-H.** Networks of DEGs either up or down-regulated in the various tissues in the indicated portions of the Venn diagram in (**A**). C1: dysregulated in CO vs. VIV but not in SO+CO vs. VIV; S1: dysregulated in SO+CO vs. VIV but not in CO vs. VIV; C2: dysregulated in CO vs. VIV but not in PL+CO vs. VIV; P2 dysregulated in PL+CO vs. VIV but not in CO vs. VIV; S3 dysregulated in SO+CO vs. VIV but not in PL+CO vs. VIV; P3 dysregulated in PL+CO vs. VIV but not in SO+CO vs. VIV. Networks were identified using Cytoscape; FDRs of individual pathways are indicated. KEGG (**C,D,G,H**) or Reactome (**B,E,F**).
