# Supplementary material for: Impact of various high fat diets on gene expression and the microbiome across the mouse intestines
Source: Sci Rep. 2023 Dec 27;13:22758. doi: 10.1038/s41598-023-49555-7 (PMC10752901; doi:10.1038/s41598-023-49555-7)
Supplement: Supplementary file 2 — Supplementary Table S1. [file 41598_2023_49555_MOESM2_ESM.pdf]

**Supplementary Table S1.**
Composition of diets and oils used in study

| **Nutrient Composition of Diets, gm %** | **Viv Chow[a]** | **CO[b]** | **SO + CO[b]** | **PL + CO[b]** |
|---|---|---|---|---|
| Protein | 23.9 | 20.1 | 20.1 | 20.1 |
| Carbohydrate | 48.7 | 53.4 | 53.4 | 53.4 |
| Fat | 5 | 21.5 | 21.5 | 21.5 |
| kcal/gm | 3.36 | 4.87 | 4.87 | 4.87 |
| Fat, kcal % | 13.4 | 40 | 40 | 40 |

| **Source of fat, gm %** | **Viv Chow[a]** | **CO** | **SO + CO** | **PL + CO** |
|---|---|---|---|---|
| Porcine animal fat | 4.5 | 0 | 0 | 0 |
| Soybean oil | 0 | 25 | 115 | 0 |
| Plenish oil | 0 | 0 | 0 | 115 |
| Coconut oil, hydrogenated | 0 | 220 | 130 | 130 |

| **Fatty acid composition of oils** | **Coconut** | **Soybean** | **Plenish** |
|---|---|---|---|
| Linoleic acid (18:2 ω6) | <0.06 | 52.9 | 7.42 |
| Oleic Acid (18:1) | 0.25 | 20.9 | 73.9 |
| Lauric Acid (12:0) | 45 | 0 | 0 |

Abbreviations: CO, coconut oil; PL, Plenish; SO, soybean oil.
[a]Purina Test Diet 5001.
[b]Diets were formulated at Research Diets, Inc
